# A standardized framework for risk-based assessment of treatment effect heterogeneity in observational healthcare databases

**DOI:** 10.1038/s41746-023-00794-y

**Published:** 2023-03-30

**Authors:** Alexandros Rekkas, David van Klaveren, Patrick B. Ryan, Ewout W. Steyerberg, David M. Kent, Peter R. Rijnbeek

**Affiliations:** 1 Department of Medical Informatics, Erasmus University Medical Center, Rotterdam, The Netherlands; 2 Department of Public Health, Erasmus University Medical Center, Rotterdam, The Netherlands; 3 Predictive Analytics and Comparative Effectiveness (PACE) Center, Institute for Clinical Research and Health Policy Studies (ICRHPS), Tufts Medical Center, Boston, MA USA; 4 Janssen Research and Development, 125 Trenton Harbourton Road, Titusville, NJ 08560 USA; 5 Department of Biomedical Informatics, Columbia University Irving Medical Center, New York, New York USA; 6 Department of Biomedical Data Sciences, Leiden University Medical Center, Leiden, The Netherlands

**Keywords:** Outcomes research, Epidemiology

## Abstract

Treatment effects are often anticipated to vary across groups of patients with different baseline risk. The Predictive Approaches to Treatment Effect Heterogeneity (PATH) statement focused on baseline risk as a robust predictor of treatment effect and provided guidance on risk-based assessment of treatment effect heterogeneity in a randomized controlled trial. The aim of this study is to extend this approach to the observational setting using a standardized scalable framework. The proposed framework consists of five steps: (1) definition of the research aim, i.e., the population, the treatment, the comparator and the outcome(s) of interest; (2) identification of relevant databases; (3) development of a prediction model for the outcome(s) of interest; (4) estimation of relative and absolute treatment effect within strata of predicted risk, after adjusting for observed confounding; (5) presentation of the results. We demonstrate our framework by evaluating heterogeneity of the effect of thiazide or thiazide-like diuretics versus angiotensin-converting enzyme inhibitors on three efficacy and nine safety outcomes across three observational databases. We provide a publicly available R software package for applying this framework to any database mapped to the Observational Medical Outcomes Partnership Common Data Model. In our demonstration, patients at low risk of acute myocardial infarction receive negligible absolute benefits for all three efficacy outcomes, though they are more pronounced in the highest risk group, especially for acute myocardial infarction. Our framework allows for the evaluation of differential treatment effects across risk strata, which offers the opportunity to consider the benefit-harm trade-off between alternative treatments.

## Introduction

Treatment effects often vary substantially across individual patients, causing overall effect estimates to be inaccurate for a significant proportion of the patients at hand^1,2^. Understanding this heterogeneity of treatment effects (HTE) has been crucial for both personalized (or precision) medicine and comparative effectiveness research, giving rise to a wide range of approaches for its discovery, evaluation and application in clinical practice. A common approach to evaluating HTE in clinical trials is through subgroup analyses. However, as these analyses are rarely adequately powered, they can lead to false conclusions of absence of HTE or exaggerate its presence^3,4^. In addition, patients differ in multiple characteristics simultaneously, resulting in much richer HTE compared to the heterogeneity explored with regular one-variable-at-a-time subgroup analyses.

Baseline risk is a summary score inherently related to treatment effect that can be used to represent the variability in patient characteristics^3,5–8^. For example, an invasive coronary procedure—compared to medical treatment—improves survival in patients with myocardial infarction at high (predicted) baseline risk but not in those at low baseline risk^9^. It has also been shown that high-risk patients with pre-diabetes benefit substantially more from a lifestyle modification program than low-risk patients^10^.

The recently proposed Predictive Approaches to Treatment effect Heterogeneity (PATH) statement provides systematic guidance on the application of risk-based methods for the assessment of HTE in randomized controlled trial (RCT) data^11,12^. After risk-stratifying patients using an existing or an internally derived prediction model, risk stratum-specific estimates of relative and absolute treatment effect are evaluated. Several methods for predictive HTE analysis have been adapted for use in observational data, but risk-based methods are still not readily available and have been highlighted as an important future research need^12^.

The Observational Health Data Science and Informatics (OHDSI) collaborative has established a global network of data partners and researchers that aim to bring out the value of health data through large-scale analytics by mapping local databases to the Observational Medical Outcomes Partnership (OMOP) Common Data Model (CDM)^13,14^. A standardized framework applying current best practices for comparative effectiveness studies within the OHDSI setting has been proposed^15^. This framework was successfully implemented in the Large-scale Evidence Generation and Evaluation across a Network of Databases for Hypertension (LEGEND-HTN) study. In this study, average effects of all first-line hypertension treatment classes were estimated for a total of 55 outcomes across a global network of nine observational databases^16^.

LEGEND-HTN found benefit for patients treated with thiazide or thiazide-like diuretics compared to angiotensin-converting enzyme (ACE) inhibitors in terms of three main outcomes of interest, i.e., acute myocardial infarction (MI), hospitalization with heart failure, and stroke. Thiazide or thiazide-like diuretics also had a better safety profile compared to ACE inhibitors which, according to that study, makes them an attractive option for first-line treatment of hypertension. However, as already pointed out, overall (average) effect estimates may not be applicable to large portions of the target population due to strong variability of important patient characteristics. A risk-based analysis of treatment effect heterogeneity can add further insights to the results of LEGEND-HTN, both in understanding how treatment effects evolve with increasing baseline outcome risk and in identifying patient subgroups, which could be targeted with a certain treatment.

Hereto, we focus on the three main outcomes of LEGEND-HTN (acute MI, hospitalization with heart failure, and stroke) and nine safety outcomes (hyponatremia, hypotension, acute renal failure, angioedema, kidney disease, cough, hyperkalemia, hypokalemia, and gastrointestinal bleeding). For our analyses, we develop a systematic framework for risk-based assessment of treatment effect heterogeneity in observational healthcare databases, extending the existing methodology from the RCT setting. The suggested framework is also implemented in an open-source, publicly available R-package. It is highly scalable and can be easily implemented across a network of observational databases mapped to OMOP-CDM.

## Results

### Overview

**Fig. 1:**
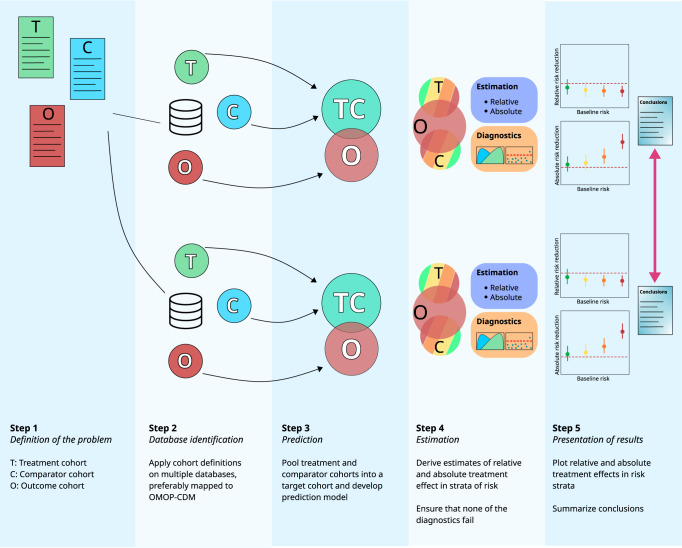
Framework overview. Illustration of the framework’s application on two observational databases, preferably mapped to OMOP-CDM.

The proposed framework defines 5 distinct steps: (1) definition of the research aim; (2) identification of the databases within which the analyses will be performed; (3) prediction of outcomes of interest; (4) estimation of absolute and relative treatment effects within risk strata; (5) presentation of the results. We developed an open-source R-package for the implementation of the proposed framework and made it publicly available (https://github.com/OHDSI/RiskStratifiedEstimation). An overview of the entire framework can be found in Fig. 1.

As a demonstration, we evaluated treatment effect heterogeneity of thiazide or thiazide-like diuretics compared to ACE inhibitors using acute MI risk quarter-specific effect estimates, both on the relative and on the absolute scale. We focused on three efficacy outcomes (acute MI, hospitalization with heart failure, and ischemic or hemorrhagic stroke) and nine safety outcomes (acute renal failure, kidney disease, cough, hyperkalemia, hypokalemia, gastrointestinal bleeding, hyponatremia, hypotension, and angioedema). We used data from three US-based claims databases.

### Step 1: General definition of the research aim

We considered the following research aim: “compare the effect of thiazide or thiazide-like diuretics ($T$) to the effect of ACE inhibitors ($C$) in patients with established hypertension with respect to 12 outcomes ($O_1, \ldots ,O_{12}$)”. The required cohorts are:

- Treatment cohort: Patients receiving any drug within the class of thiazide or thiazide-like diuretics with at least one year of follow-up before treatment initiation and a recorded hypertension diagnosis within that year.
- Comparator cohort: Patients receiving any drug within the ACE inhibitor class with at least one year of follow-up before treatment initiation and a recorded hypertension diagnosis within that year.
- Outcome cohorts: We considered three efficacy and nine safety outcome cohorts. These were patients in the database with a diagnosis of: acute MI; hospitalization with heart failure; ischemic or hemorrhagic stroke (efficacy outcomes); acute renal failure; kidney disease; cough; hyperkalemia; hypokalemia; gastrointestinal bleeding; hyponatremia; hypotension; angioedema (safety outcomes).

All cohort definitions were identical to the ones used in the multinational LEGEND-HTN study^16^. More information can be found in the Supplementary Results (Sections A and B) and Supplementary Tables 1–19.

### Step 2: Identification of the databases

For our demonstration we used data from three US claims databases, namely IBM® MarketScan® Commercial Claims and Encounters (CCAE), IBM® MarketScan® Multi-State Medicaid (MDCD), and IBM® MarketScan® Medicare Supplemental Beneficiaries (MDCR). More information on the included databases can be found in Supplementary Results Section D. Our analyses included a total of 355,826 (CCAE), 54,835 (MDCD), and 37,882 (MDCR) patients initiating treatment with thiazide or thiazide-like diuretics and 930,629 (CCAE), 106,492 (MDCD), and 105,852 (MDCR) patients initiating treatment with ACE inhibitors (Table 1). Patient characteristics are available in Supplementary Tables 20–22. Adequate numbers of patients were included in all strata of predicted acute MI risk (Supplementary Table 23).

**Table 1:** Sample sizes. Number of patients, person years and events for the three efficacy outcomes of the study across the three databases after excluding patients with prior outcomes.

	Thiazides or thiazide-like diuretics			Ace inhibitors		
Outcome	Patients	Person years	Outcomes	Patients	Person years	Outcomes
CCAE						
Acute myocardial infarction	355,826	204,593	405	930,369	584,167	1813
Hospitalization with heart failure	355,528	204,451	389	930,629	584,541	1492
Stroke	354,446	203,792	425	923,604	579,736	1636
MDCD						
Acute myocardial infarction	54,835	21,440	76	106,492	51,481	440
Hospitalization with heart failure	54,354	21,290	212	105,005	50,878	835
Stroke	54,259	21,179	149	104,410	50,334	562
MDCR						
Acute myocardial infarction	37,882	24,642	161	105,852	74,990	732
Hospitalization with heart failure	37,617	24,509	277	105,134	74,654	1196
Stroke	37,248	24,267	261	102,502	72,705	977

### Step 3: Prediction

We internally developed separate prediction models for 2-year acute MI risk in each of the three databases. The prediction models were fitted on the propensity score-matched (1:1) subset of the entire study population, using a caliper of 0.2 and after excluding patients having the outcome at any time prior to treatment initiation. We considered a large set of candidate predictors containing patients’ demographic information (age, sex), disease and medication history, and the Charlson comorbidity index (Romano adaptation) measured in the year prior to treatment initiation. As all three databases are mapped to OMOP-CDM, coding of all predictors was uniform across databases. This enables the development of the prediction models for acute MI risk in a uniform fashion across databases. However, due to the differences in data capture among databases, we cannot expect that all covariates will be present in all databases. We developed the prediction models using LASSO logistic regression with 3-fold cross validation for hyper-parameter selection. In Supplementary Table 24 we show the available sample sizes on which the prediction models were developed, while in Supplementary Tables 25–27 we show the 20 selected covariates with the largest coefficients in each database.

The models had moderate discriminative ability (internally validated) in CCAE and MDCD and lower discriminative ability in MDCR (Table 2).

**Table 2:** Prediction performance. Discriminative ability (c-statistic) of the derived prediction models for acute MI in the matched set (development set), the treatment cohort, the comparator cohort, and the entire population in CCAE, MDCD, and MDCR. Values in parentheses are cross-validated 95% confidence intervals. Matched population is the propensity score-matched subset in each database on which the prediction models were developed. Treatment population is the set of patients receiving thiazide or thiazide-like diuretics in each database, while comparator population is the set of patients receiving ACE inhibitors. Finally, entire population refers to the combined set of treatment and comparator patients.

Population	CCAE	MDCD	MDCR
Matched	0.73 (0.71, 0.74)	0.76 (0.73, 0.79)	0.65 (0.62, 0.68)
Treatment	0.73 (0.71, 0.75)	0.82 (0.77, 0.86)	0.66 (0.62, 0.70)
Comparator	0.70 (0.67, 0.71)	0.74 (0.71, 0.76)	0.66 (0.64, 0.68)
Entire population	0.71 (0.70, 0.72)	0.76 (0.74, 0.78)	0.66 (0.64, 0.68)

### Step 4: Estimation

In each database, we used patient-level predictions of the internally derived acute MI risk prediction model to stratify the patients into three acute MI risk groups RG-1, RG-2, and RG-3 (patients below 1% risk, patients between 1% and 1.5% risk, and patients above 1.5% risk). Within risk groups, in order to account for observed confounding, we further stratified the patients into five propensity score strata. Propensity score models were developed within each risk group separately using the same approach as in step 3 (LASSO logistic regression with a large set of predefined covariates). Risk group-specific relative treatment effects were estimated by averaging over the hazard ratio estimates derived from Cox regression models fitted in each propensity score stratum. Similarly, risk group-specific absolute treatment effects were estimated by averaging over the differences in Kaplan-Meier estimates in each propensity score stratum at 2 years after treatment initiation.

**Fig. 2:**
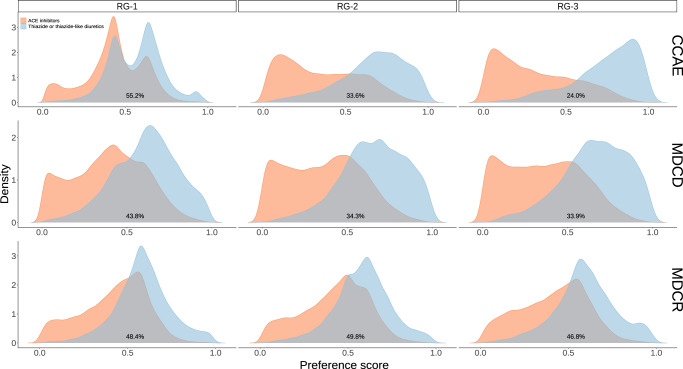
Preference score distributions within strata of predicted acute MI risk. RG-1 represents patients with acute MI risk lower than 1%; RG-2 represents patients with acute MI risk between 1% and 1.5%; RG-3 represents patients with acute MI risk larger than 1.5%. The preference score is a transformation of the propensity score that adjusts for prevalence differences between populations. The percentages in each figure represent the amount of preference score overlap between treatment arms. Higher overlap of the preference score distributions indicates that patients in the target and the comparator cohorts are more similar in terms of the predicted probability of receiving treatment (thiazide or thiazide-like diuretics).

**Fig. 3:**
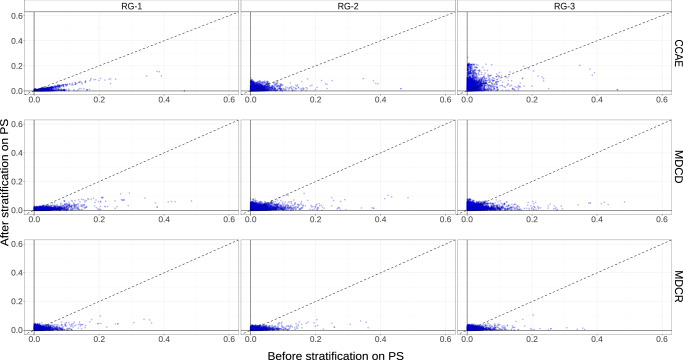
Covariate balance. Patient characteristic balance for thiazide or thiazide-like diuretics and ACE inhibitors before and after stratification on the propensity scores. RG-1 represents patients with acute MI risk lower than 1%; RG-2 represents patients with acute MI risk between 1% and 1.5%; RG-3 represents patients with acute MI risk larger than 1.5%. Each point represents the standardized difference of means for a single covariate before (x-axis) and after (y-axis) stratification. A commonly used rule of thumb suggests that standardized mean differences above 0.1 after propensity score adjustment indicate insufficient covariate balance.

In all databases we found adequate overlap of the propensity score distributions across the risk groups, except for high-risk patients in CCAE (acute MI risk above 1.5%). Hence, the propensity scores should be able to adjust for observed confounding, except for high-risk CCAE patients (Fig. 2). The covariate balance plots comparing covariate standardized mean differences before and after adjustment with the propensity scores confirmed strong imbalances for CCAE patients with acute MI predicted risk above 1.5% (Fig. 3). Owing to very limited overlap of the preference score distributions (Fig. 2) and persisting imbalances after stratification on the propensity scores (Fig. 3), we do not present the results for patients at risk above 1.5% for acute MI in CCAE. Additionally, a small number of characteristics remained slightly imbalanced even after stratification on the propensity scores for the two lower acute MI risk groups of MDCD (Fig. 3). Therefore, results from analyses in this database should be interpreted with caution.

**Fig. 4:**
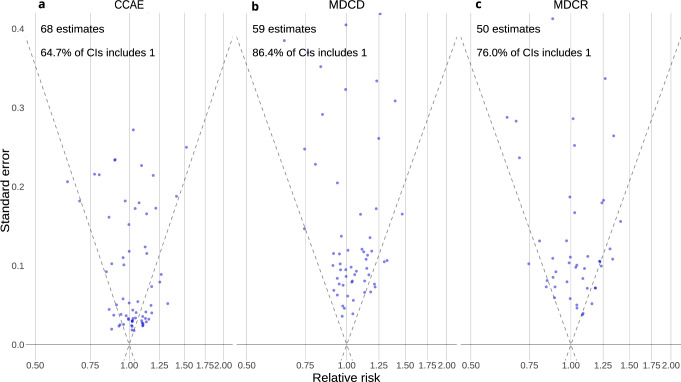
Systematic error. Effect size estimates for the negative controls (true hazard ratio = 1) in **a** CCAE, **b** MDCD, and **c** MDCR databases. Estimates below the diagonal dashed lines are statistically significant (different from the true effect size; alpha = 0.05). A well-calibrated estimator should include the true effect size within the 95% confidence interval, 95% of times.

Finally, the distribution of the estimated relative risks with regard to a total of 76 negative control outcomes (Supplementary Results, Section C) showed no evidence of residual confounding, except for CCAE (Fig. 4)^17–19^. Hazard ratios for CCAE (Fig. 4, panel a) were often significantly larger than 1 (true effect size). This suggests significant negative effects of thiazide or thiazide-like diuretics compared to ACE inhibitors on causally unrelated outcomes, indicating unresolved differences between the two treatment arms. Therefore, results from CCAE should be interpreted with caution, as residual confounding may still be present, despite our propensity score adjustment. The results of the risk-stratified negative control analyses for each database can be found in Supplementary Figs. 1–3.

### Step 5: Presentation of results

On average, thiazide or thiazide-like diuretics were beneficial compared to ACE inhibitors for all outcomes, except for hospitalization with heart failure in CCAE and stroke in MDCD (Table 3). The hazard ratios are in line with, but not equal to, those reported in the LEGEND-HTN study, mainly because of restricting time at risk to two years.

**Table 3:** Relative effect estimates. Hazard ratio estimates for the overall treatment effect of thiazide or thiazide-like diuretics compared to ACE inhibitors. Values in brackets are 95% confidence intervals.

Outcome	CCAE	MDCD	MDCR
Acute myocardial infarction	0.86 (0.77, 0.97)	0.60 (0.46, 0.77)	0.82 (0.68, 0.98)
Hospitalization with heart failure	0.99 (0.88, 1.12)	0.84 (0.71, 0.99)	0.83 (0.72, 0.95)
Stroke	0.87 (0.78, 0.97)	0.87 (0.71, 1.06)	0.90 (0.78, 0.95)

**Fig. 5:**
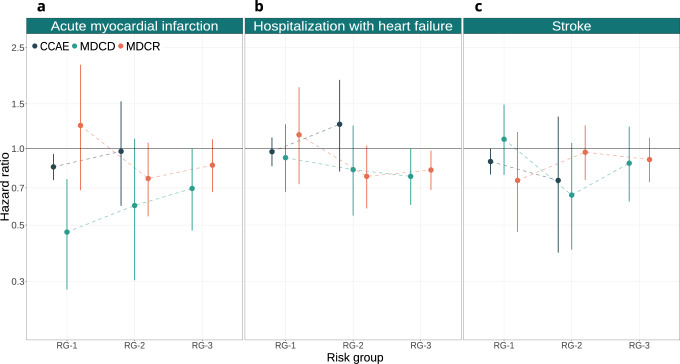
Relative treatment effects for main outcomes. Treatment effect heterogeneity for the main outcomes on the relative scale (hazard ratios) of thiazide or thiazide-like diuretics compared to ACE inhibitors within strata of predicted acute MI risk. In **a** we present treatment effects on the relative scale for acute MI within groups of predicted acute MI risk across all three databases. In **b** we present treatment effects on the relative scale for hospitalization with heart failure within groups of predicted acute MI risk across all three databases. In **c** we present treatment effects on the relative scale for stroke (both ischemic and hemorrhagic) within groups of predicted acute MI risk across all three databases. RG-1 represents the group of patients with acute MI risk below 1%; RG-2 represents the group of patients with acute MI risk between 1% and 1.5%; RG-3 represents the group of patients with acute MI risk larger than 1.5%. Hazard ratios estimated in CCAE, MDCD, and MDCR are represented by blue, green, and orange circles, respectively. The bars represent 95% confidence intervals. Values below 1 favor thiazide or thiazide-like diuretics, while values above 1 favor ACE inhibitors.

For the primary outcomes (acute MI, hospitalization with heart failure and stroke) relative treatment effect estimates of thiazide or thiazide-like diuretics versus ACE inhibitors varied substantially across risk groups, but no clear trends indicating an association between risk and relative treatment effect estimates were observed (Fig. 5).

For acute MI, hazard ratios showed an increasing trend with increasing baseline acute MI risk in MDCD and CCAE, implying larger benefit on the relative scale for patients in the lower risk groups. This was less pronounced in MDCR (Fig. 5; panel a). For hospitalization with heart failure, hazard ratios were similar across all acute MI risk strata in MDCD, with a slightly decreasing trend favoring thiazide or thiazide-like diuretics (Fig. 5; panel b). In MDCR, these hazard ratios were very similar to MDCD for patients at acute MI risk higher than 1%. For patients below 1% acute MI risk, hazard ratios were close to 1 (negligible relative treatment effects) in all three databases. Finally, for stroke, the hazard ratios indicated a beneficial effect of thiazide or thiazide-like diuretics in all databases, but we found no clear trends in hazard ratios across acute MI risk groups (Fig. 5; panel c).

**Fig. 6:**
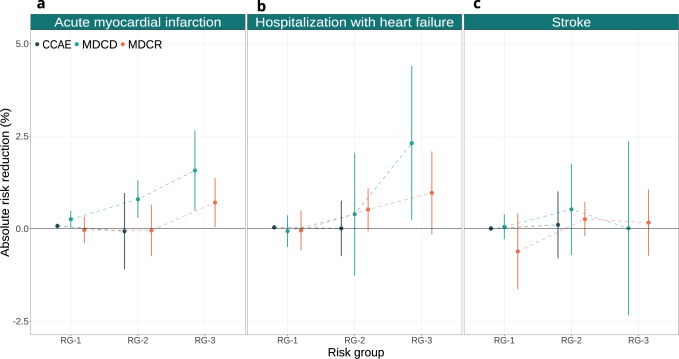
Absolute treatment effects for main outcomes. Treatment effect heterogeneity for the main outcomes on the absolute scale of thiazide or thiazide-like diuretics compared to ACE inhibitors within strata of predicted acute MI risk. In **a** we present treatment effects on the absolute scale for acute MI within groups of predicted acute MI risk across all three databases. In **b** we present treatment effects on the absolute scale for hospitalization with heart failure within groups of predicted acute MI risk across all three databases. In **c** we present treatment effects on the absolute scale for stroke (both ischemic and hemorrhagic) within groups of predicted acute MI risk across all three databases. RG-1 represents the group of patients with acute MI risk below 1%; RG-2 represents the group of patients with acute MI risk between 1% and 1.5%; RG-3 represents the group of patients with acute MI risk larger than 1.5. Absolute treatment effects estimated in CCAE, MDCD, and MDCR are represented by blue, green, and orange circles, respectively. The bars represent 95% confidence intervals. Values above 0 favor thiazide or thiazide-like diuretics, while values below 0 favor ACE inhibitors.

Absolute treatment effects (risk reduction) for acute MI and hospitalization with heart failure tended to increase with increasing acute MI risk (Fig. 6; panels a and b). This was most evident in MDCD, where the absolute benefits for acute MI were 0.25% (0.03% to 0.48%; 95% CI) and 1.57% (0.49% to 2.65%; 95% CI) in the lowest and the highest acute MI risk group, respectively. Similarly, in MDCR these absolute benefits were −0.04% (−0.40% to 0.32%; 95% CI) and 0.70% (0.04% to 1.37%; 95% CI), respectively. For hospitalization with heart failure, these absolute benefits were −0.07% (−0.50% to 0.36%; 95% CI) and 2.31% (0.22% to 4.39%; 95% CI), respectively, in MDCD and −0.05% (−0.59% to 0.49%; 95% CI) and 0.97% (−0.16% to 2.09%; 95% CI), respectively, in MDCR. In CCAE, we found negligible treatment effects on the absolute scale for all three outcomes. Finally, for stroke, the differences on the absolute scale were small in all risk groups and databases (Fig. 6; panel c).

**Fig. 7:**
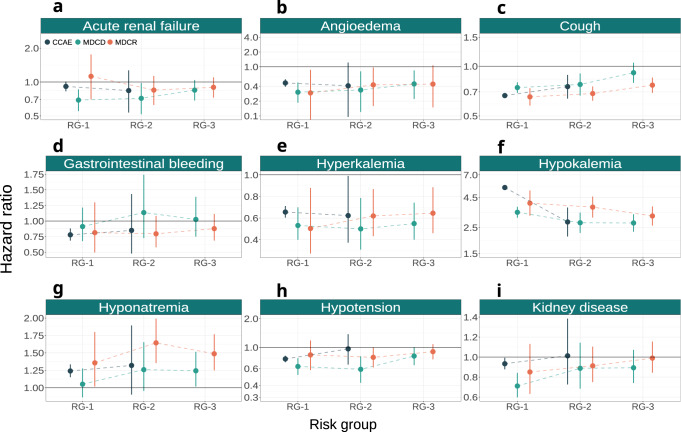
Relative treatment effects for safety outcomes. Treatment effect heterogeneity for the safety outcomes on the relative scale (hazard ratios) of thiazide or thiazide-like diuretics compared to ACE inhibitors within strata of predicted acute MI risk. Panels present treatment effects on the relative scale for **a** acute renal failure, **b** angioedema, **c** cough, **d** gastrointestinal bleeding, **e** hyperkalemia, **f** hypokalemia, **g** hyponatremia, **h** hypotension, and **i** kidney disease within groups of predicted acute MI risk across all three databases. RG-1 represents the group of patients with acute MI risk below 1%; RG-2 represents the group of patients with acute MI risk between 1% and 1.5%; RG-3 represents the group of patients with acute MI risk larger than 1.5%. Hazard ratios estimated in CCAE, MDCD, and MDCR are represented by blue, green, and orange circles, respectively. Bars represent 95% confidence intervals. Values below 1 favor thiazide or thiazide-like diuretics, while values above 1 favor ACE inhibitors.

Across all databases and all risk groups (Fig. 7), thiazide or thiazide-like diuretics reduced the risk for angioedema, cough, hyperkalemia, and hypotension, but were associated with increased risk of hypokalemia and hyponatremia. For cough and hypokalemia, the relative treatment effect tended to decrease with increasing MI risk (hazard ratios moving closer to 1).

**Fig. 8:**
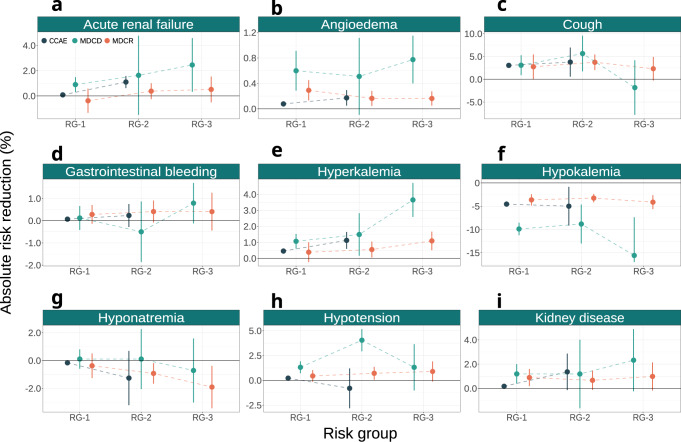
Absolute treatment effects for safety outcomes. Treatment effect heterogeneity for the safety outcomes on the absolute scale of thiazide or thiazide-like diuretics compared to ACE inhibitors within strata of predicted acute MI risk. Panels present treatment effects on the absolute scale for **a** acute renal failure, **b** angioedema, **c** cough, **d** gastrointestinal bleeding, **e** hyperkalemia, **f** hypokalemia, **g** hyponatremia, **h** hypotension, and **i** kidney disease within groups of predicted acute MI risk across all three databases. RG-1 represents the group of patients with acute MI risk below 1%; RG-2 represents the group of patients with acute MI risk between 1% and 1.5%; RG-3 represents the group of patients with acute MI risk larger than 1.5%. Absolute treatment effects estimated in CCAE, MDCD, and MDCR are represented by blue, green, and orange circles, respectively. The bars represent 95% confidence intervals. Values above 0 favor thiazide or thiazide-like diuretics, while values below 0 favor ACE inhibitors.

The absolute benefit for angioedema of thiazide or thiazide-like diuretics was negligible, despite the large treatment effect estimated on the relative scale (Fig. 8; panel b). The absolute risk increase of hypokalemia was large with thiazide or thiazide diuretics—as expected based on the effect estimates on the relative scale—across all risk strata (Fig. 8; panel f). This effect remained relatively constant across acute MI risk groups in MDCR, fluctuating between −4.13% and −3.25%. Similar effects on the absolute scale were observed in CCAE, where effect estimates were close to −5% for all patients below 1.5% risk of acute MI. A much larger hypokalemia risk increase with thiazide or thiazide-like diuretics was observed in MDCD, where the absolute effect estimates evolved from −9.89% (−11.23% to −8.54%; 95% CI) in patients below 1% acute MI risk to −15.58% (−23.78% to −7.38%; 95% CI) in patients above 1.5% acute MI risk. The absolute benefit estimates of thiazide or thiazide-like diuretics for cough ranged between 3.05% and 3.77% in CCAE, and between 2.32% and 3.73% in MDCR (Fig. 8; panel c). In MDCD, we observed a small risk increase of cough with thiazide or thiazide-like diuretics in patients at high acute MI baseline risk (−1.82% with a 95% CI from −7.82% to 4.17%). Finally, we observed a small risk increase of hyponatremia with thiazide or thiazide diuretics, which was more substantial in patients with high acute MI risk in MDCR (−1.91% with a 95% CI from −3.43% to −0.38%).

### Interpretation

The overall benefits of thiazide or thiazide-like diuretics compared to ACE inhibitors that were observed in MDCR, in terms of acute MI and hospitalization with heart failure, were mainly driven by patients with predicted acute MI risk above 1.5%. Even in MDCD, where benefit on the absolute scale was observed across all acute MI risk strata, treatment effects were much larger in patients with predicted acute MI risk above 1.5%. In CCAE, where the majority of the patients had a predicted acute MI risk below 1%, we found negligible treatment effects. This provides further support for the similarity of the effect of thiazide or thiazide-like diuretics compared to ACE inhibitors in patients at low risk of acute MI.

Even though LEGEND-HTN found beneficial effects of thiazide or thiazide-like diuretics over ACE inhibitors in terms of several safety outcomes, there are still safety concerns when prescribing thiazide or thiazide-like diuretics. The hypokalemia and hyponatremia risk increase with thiazide or thiazide-like diuretics was not negligible in any of the acute MI risk strata. On the other hand, ACE inhibitor-related cough risk increase was also present in all databases and acute MI risk groups. Provided that absolute benefits of thiazide or thiazide-like diuretics for the main outcomes (acute MI, hospitalization with heart failure, and stroke) were mainly observed in patients at high acute MI risk, the prescribing physician has to carefully weigh benefits and harms for individual patients.

Note that any conclusions drawn are for demonstration purposes only and should be interpreted under this very limited setting.

### Sensitivity analyses

As a sensitivity analysis, we evaluated treatment effect heterogeneity of thiazide or thiazide-like diuretics compared to ACE inhibitors in patients with or without prior cardiovascular disease. We defined the set of patients without prior cardiovascular disease as the patients that had no occurrence in their medical history of any of the following conditions: heart valve disorder or transplanted heart valve, coronary artery disease, cardiac dysfunction, heart block, unstable angina, atrial fibrillation, myocardial infarction, ventricular arrhythmia or cardiac arrest, ischemic heart disease, myocarditis or pericarditis, cardiomyopathy, cardiomegaly, heart failure, or stroke (ischemic or hemorrhagic). If patients had any of these conditions recorded in their medical history, they were assigned to the group with prior cardiovascular disease. We repeated our analyses using the exact same settings for both groups of patients.

In patients without prior cardiovascular disease, the estimates of the relative effect of thiazide or thiazide-like diuretics compared to ACE inhibitors on acute MI were similar to the original analyses—hazard ratios 0.90 (0.79 to 1.02; 95% CI), 0.52 (0.36 to 0.74; 95% CI), and 0.83 (0.65 to 1.05; 95% CI) in CCAE, MDCD, and MDCR respectively. In patients with prior cardiovascular disease the effect of thiazide or thiazide-like diuretics was stronger in CCAE—hazard ratio 0.73 (0.55 to 0.95; 95% CI)—but weaker in MDCD and MDCR—hazard ratios 0.78 (0.51 to 1.16; 95% CI) and 0.88 (0.66 to 1.15; 95% CI), respectively. In both sets of sensitivity analyses, risk-stratified results showed trends comparable to the original analysis (Supplementary Figs. 4–11).

## Discussion

In this study we develop a risk-based framework for the assessment of treatment effect heterogeneity in large observational databases. Our framework fills a gap identified in the literature after the development of guidelines for performing such analyses in the RCT setting^11,12^. As an additional contribution we provide the software for implementing this framework in practice and make it publicly available. We made our software compatible to databases mapped to OMOP-CDM, which allows researchers to easily implement our framework in a global network of healthcare databases. In our case study we demonstrate the use of our framework for the evaluation of treatment effect heterogeneity of thiazide or thiazide-like diuretics compared to ACE inhibitors on three efficacy and nine safety outcomes. We propose that this framework is implemented any time treatment effect estimation in high-dimensional observational data is undertaken.

In recent years, several methods for the analysis of treatment effect heterogeneity have been developed in the RCT setting^20^. However, low power and restricted prior knowledge on the mechanisms of variation in treatment effect are often inherent in RCTs, which are usually adequately powered only for the analysis of the primary outcome. Observational databases contain a large amount of information on treatment assignment and outcomes of interest, while also capturing key patient characteristics. They contain readily available data on patient sub-populations of interest on which no RCT has focused before either due to logistical or ethical reasons. However, observational databases can be susceptible to biases, poorly measured outcomes and missingness, which may obscure true HTE or falsely indicate it when there is none^21^. Therefore, inferences on both overall treatment effect estimates and HTE need to rely on strong—often unverifiable—assumptions, despite the advancements and guidance on best practices. When evaluating treatment effect heterogeneity using a risk-based approach these issues may be compounded, mainly because of the risk of conflating confounding and effect modification. Well-designed observational studies on average replicate RCT results, even though often differences in magnitude may occur^22^. Our framework is in line with the recently suggested paradigm of high-throughput observational studies using consistent and standardized methods for improving reproducibility in observational research^19^. However, more empirical research comparing analyses of observational data and RCTs is required to assess the conditions under which different approaches for evaluating treatment effect heterogeneity provide credible results. Our software package can help support this research.

Our framework highlights the scale dependency of HTE and how it relates to baseline risk. Treatment effect is mathematically determined by baseline risk, if we assume a constant non-zero effect size^23^. Patients with low baseline risk can only experience minimal benefits, before their risk is reduced to zero. In contrast, high-risk patients can potentially have much larger absolute benefits. This becomes evident when evaluating the safety of thiazide or thiazide-like diuretics on angioedema and cough, both adverse events linked to treatment with ACE inhibitors. For angioedema, the substantial relative risk increase with ACE inhibitors only translated in a small risk increase on the absolute scale due to the limited baseline angioedema risk. Conversely, despite the small relative cough risk increase of ACE inhibitors, the large baseline cough risk resulted in larger absolute risk differences, compared to the other considered outcomes.

For patients with comorbidities the Guidelines of the American College of Cardiology often recommend initiation of treatment with ACE inhibitors, e.g., for patients with stable ischemic heart disease or patients with preserved ejection fraction^24^. Since these are patients with more severe medical conditions there may be a potential interaction of baseline acute MI risk with the propensity of receiving a thiazide or a thiazide-like diuretic. We do not formally test for that interaction, however, we observed that with increasing acute MI baseline risk, the overlap of the propensity score distributions decreases and the propensity score distributions for each treatment arm become more skewed, especially in CCAE and MDCD (Fig. 2). This could potentially result in unobserved confounding being present even after propensity score adjustment. Indeed, in CCAE, negative control analyses showed evidence of residual confounding and therefore results should be interpreted with caution. In risk-stratified negative control analyses we observed more evidence of residual confounding in patients with higher acute MI risk, which was, however, not identified in the other two databases.

The application of our framework in the case study is for demonstration purposes and there are several limitations to its conclusions. First, risk groups defined in each database were not defined using a universal prediction model, but using internally developed prediction models in each database. Future research could explore model combination or transfer learning methods for the development of universal risk prediction models. Second, death could be a competing risk. We could expand our framework in the future to potentially support sub-distribution hazard ratios and cumulative incidence reductions. Third, we only used the databases readily available to us and not all the available databases mapped to OMOP-CDM. Therefore, the generalizability of our results still needs to be explored in future studies. These studies should also address the particular aspects of the databases at hand, such as their sampling frame, the completeness of the data they capture and many other aspects that were not assessed in our demonstration. Fourth, we did not correct for multiplicity when presenting the results. We are interested in presenting trends in the data rather than detecting specific subgroups with significant treatment effects. The implementation of our framework, however, generates all the relevant output required for a researcher to correct for multiple testing, if that is required.

In conclusion, the case study demonstrates the feasibility of our framework for risk-based assessment of treatment effect heterogeneity in large observational data. It is easily applicable and highly informative whenever treatment effect estimation in high-dimensional observational data is of interest.

## Methods

### Step 1: General definition of the research aim

The typical research aim is: “to compare the effect of treatment to a comparator treatment in patients with a disease with respect to outcomes $O_1, \ldots ,O_n$”.

We use a comparative cohort design. This means that at least three cohorts of patients need to be defined at this stage of the framework:

- A single treatment cohort ($T$), which includes patients with disease receiving the target treatment of interest.
- A single comparator cohort ($C$), which includes patients with disease receiving the comparator treatment.
- One or more outcome cohorts ($O_1, \ldots ,O_n$) that contain patients developing the outcomes of interest

### Step 2: Identification of the databases

Including in our analyses multiple databases representing the population of interest potentially increases the generalizability of results. Furthermore, the cohorts should preferably have adequate sample size with adequate follow-up time to ensure precise effect estimation, even within smaller risk strata. Other relevant issues such as the depth of data capture (the precision at which measurements, lab tests, conditions are recorded) and the reliability of data entry should also be considered.

In our analyses, we used data from IBM® MarketScan® Commercial Claims and Encounters (CCAE), IBM® MarketScan® Medicaid (MDCD), and IBM® MarketScan® Medicare Supplemental Beneficiaries (MDCR). The New England Institutional Review Board (IRB) has determined that studies conducted in these databases are exempt from study-specific IRB review, as these studies do not qualify as human subjects research.

### Step 3: Prediction

For our risk-based approach to adequately evaluate treatment effect heterogeneity, a well performing prediction model assigning patient-level risk for the outcome of interest needs to be available, either from literature or internally developed from the data at hand. For internally developing a risk prediction model we adopt a standardized framework focused on observational data that ensures adherence to existing guidelines^25–27^. We use the derived prediction model to separate the patient population into risk strata, within which treatment effects on both the relative and the absolute scale will be assessed.

For the development of the risk prediction model, we first need to define a target cohort of patients, i.e., the set of patients on whom the prediction model will be developed. In our case, the target cohort is generated by pooling the already defined treatment and comparator cohorts. We develop the prediction model on the propensity score-matched (1:1) subset of the pooled sample to avoid differentially fitting between treatment arms, thus introducing spurious interactions with treatment^28,29^. We also need to define a set of patients that experience the outcome of interest, i.e., the outcome cohort. Finally, we need to decide the time frame within which the predictions will be carried out, i.e., the patients’ time at risk. Subsequently, we can develop the prediction model.

It is important that the prediction models display good discriminative ability to ensure that risk-based subgroups are accurately defined. A performance overview of the derived prediction models including discrimination and calibration both in the propensity score-matched subset, the entire sample and separately for treated and comparator patients should also be reported.

### Step 4: Estimation

We estimate treatment effects (both on the relative and the absolute scale) within risk strata defined using the prediction model of step 3. We often consider four risk strata, but fewer or more strata can be considered depending on the available power for accurately estimating stratum-specific treatment effects. Effect estimation may be focused on the difference in outcomes for a randomly selected person from the risk stratum (average treatment effect) or for a randomly selected person from the treatment cohort within the risk stratum receiving the treatment under study (average treatment effect on the treated).

Any appropriate method for the analysis of relative and absolute treatment effects can be considered, as long as the this is done consistently in all risk strata. Common statistical metrics are odds ratios or hazard ratios for relative scale estimates and differences in observed proportions or differences in Kaplan-Meier estimates for absolute scale estimates, depending on the problem at hand. We estimate propensity scores within risk strata which we then use to match patients from different treatment cohorts or to stratify them into groups with similar propensity scores or to weigh each patient’s contribution to the estimation process^30^.

Prior to analyzing results, it is crucial to ensure that all diagnostics are passed in all risk strata. The standard diagnostics we carry out include analysis of the overlap of propensity score distributions and calculation of standardized mean differences of the covariates before and after propensity score adjustment. Finally, we use effect estimates for a large set of negative control outcomes—i.e., outcomes known to not be related with any of the exposures under study—to evaluate the presence of residual confounding not accounted for by propensity score adjustment^17–19^.

### Step 5: Presentation of results

In the presence of a positive treatment effect and a well-discriminating prediction model we expect an increasing pattern of the differences in the absolute scale, even if treatment effects remain constant on the relative scale across risk strata. Owing to this scale-dependence of treatment effect heterogeneity, results should be assessed both on the relative and the absolute scale.

### Reporting summary

Further information on research design is available in the Nature Research Reporting Summary linked to this article.

## Supplementary information

Supplemental Material

REPORTING SUMMARY

## Data Availability

The data that support the findings of this study are available from IBM® MarketScan® but restrictions apply to the availability of these data, which were used under license for the current study, and so are not publicly available. Data are however available from the authors upon reasonable request and with permission of IBM® MarketScan®. Please contact Peter R. Rijnbeek with any data-related requests.

## Code availability

Code for the extraction of the cohorts is available at https://github.com/ohdsi/Legend. Source code or the execution of the study is available at https://github.com/mi-erasmusmc/HteFramework. The source code for the R-package that implements our framework is available at https://github.com/ohdsi/RiskStratifiedEstimation.
