# Supplementary material for: A standardized framework for risk-based assessment of treatment effect heterogeneity in observational healthcare databases
Source: NPJ Digit Med. 2023 Mar 30;6:58. doi: 10.1038/s41746-023-00794-y (PMC10060247; doi:10.1038/s41746-023-00794-y)
Supplement: Supplementary file 1 — Supplemental Material [file 41746_2023_794_MOESM1_ESM.pdf]

Supplementary material

Contents

SUPPLEMENTARY RESULTS 2
Section A. Exposure cohorts 2
First-line new user thiazide or thiazide-like diuretics 2
First-line new user ACE inhibitors 3
Acute Myocardial Infarction 4
Hospitalization with heart failure 4
Stroke (ischemic or hemorrhagic) events 5
Acute renal failure events 6
Angioedema events 6
Cough events 7
Gastrointestinal bleeding events 8
Hyponatremia events 9
Hyperkalemia events 9
Hypokalemia events 10
Hypotension events 10
Kidney disease events 11
Section C. Negative control outcomes 13
Section D. Database descriptions 15
SUPPLEMENTARY TABLES 16
SUPLLEMENTARY FIGURES 48
SUPPLEMENTARY REFERENCES 60

SUPPLEMENTARY RESULTS

Section A. Exposure cohorts

First-line new user thiazide or thiazide-like diuretics

Initial Event Cohort

People having any of the following:

- a drug exposure of Thiazide or thiazide-like diuretics (see Supplementary Table 3)
  - for the first time in the person's history

with continuous observation of at least 365 days prior and 0 days after event index date, and limit initial events to: earliest event per person.

For people matching the Primary Events, include:

Having all of the following criteria:

- exactly 0 occurrences of a drug exposure of Hypertension drugs (see Supplementary Table 2) where event starts between all days Before and 1 days Before index start date
- and at least 1 occurrences of a condition occurrence of Hypertensive disorder (see Supplementary Table 1) where event starts between 365 days Before and 0 days After index start date
- and exactly 1 distinct occurrences of a drug era of Hypertension drugs (see Supplementary Table 2) where event starts between 0 days Before and 7 days After index start date

Limit cohort of initial events to: earliest event per person.

Limit qualifying cohort to: earliest event per person.

End Date Strategy

Custom Drug Era Exit Criteria

This strategy creates a drug era from the codes found in the specified concept set. If the index event is found within an era, the cohort end date will use the era's end date. Otherwise, it will use the observation period end date that contains the index event.

Use the era end date of Thiazide or thiazide-like diuretics (see Supplementary Table 3)

- allowing 30 days between exposures
- adding 0 days after exposure end

Cohort Collapse Strategy:
Collapse cohort by era with a gap size of 0 days.

First-line new user ACE inhibitors

Initial Event Cohort

People having any of the following:

- a drug exposure of ACE inhibitors (see Supplementary Table 4)
  - for the first time in the person's history

with continuous observation of at least 365 days prior and 0 days after event index date, and limit initial events to: earliest event per person.

For people matching the Primary Events, include:

Having all of the following criteria:

- exactly 0 occurrences of a drug exposure of Hypertension drugs (see Supplementary Table 2) where event starts between all days Before and 1 days Before index start date
- and at least 1 occurrences of a condition occurrence of Hypertensive disorder (see Supplementary Table 1) where event starts between 365 days Before and 0 days After index start date
- and exactly 1 distinct occurrences of a drug era of Hypertension drugs (see Supplementary Table 2) where event starts between 0 days Before and 7 days After index start date

Limit cohort of initial events to: earliest event per person.

Limit qualifying cohort to: earliest event per person.

End Date Strategy

Custom Drug Era Exit Criteria
This strategy creates a drug era from the codes found in the specified concept set. If the index event is found within an era, the cohort end date will use the era's end date. Otherwise, it will use the observation period end date that contains the index event.

Use the era end date of ACE inhibitors (see Supplementary Table 4)

- allowing 30 days between exposures
- adding 0 days after exposure end
- using days supply and exposure end date for exposure duration.

Cohort Collapse Strategy:
Collapse cohort by era with a gap size of 0 days.

Section B. Outcome cohorts

Acute Myocardial Infarction

Initial Event Cohort

People having any of the following:

- a condition occurrence of Acute myocardial Infarction (see Supplementary Table 6)

with continuous observation of at least 0 days prior and 0 days after event index date, and limit initial events to: all events per person.

For people matching the Primary Events, include:

Having any of the following criteria:

- at least 1 occurrences of a visit occurrence of Inpatient or ER visit (see Supplementary Table 5) where event starts between all days Before and 0 days After index start date and event ends between 0 days Before and all days After index start date

Limit cohort of initial events to: all events per person.

Limit qualifying cohort to: all events per person.

End Date Strategy

Date Offset Exit Criteria
This cohort definition end date will be the index event's start date plus 7 days

Cohort Collapse Strategy:
Collapse cohort by era with a gap size of 180 days.

Hospitalization with heart failure

Inpatient or ER visits with heart failure condition record; all qualifying inpatient visits occurring > 7 days apart are considered independent episodes

Initial Event Cohort

People having any of the following:

- a visit occurrence of Inpatient or ER visit (see Supplementary Table 5)
    having one of the following:

- at least 1 occurrences of a condition occurrence of Heart Failure (see Supplementary Table 7) where event starts between 0 days Before and all days After index start date and event starts between all days Before and 0 days After index end date

with continuous observation of at least 0 days prior and 0 days after event index date, and limit initial events to: all events per person.

Limit qualifying cohort to: all events per person.

End Date Strategy

Date Offset Exit Criteria
This cohort definition end date will be the index event's end date plus 0 days

Cohort Collapse Strategy:
Collapse cohort by era with a gap size of 7 days.

Stroke (ischemic or hemorrhagic) events

Stroke (ischemic or hemorrhagic) condition record during an inpatient or ER visit; successive records with > 180 day gap are considered independent episodes

Initial Event Cohort

People having any of the following:

- a condition occurrence of Stroke ischemic or hemorrhagic (see Supplementary Table 8)

with continuous observation of at least 0 days prior and 0 days after event index date, and limit initial events to: all events per person.

For people matching the Primary Events, include:

Having any of the following criteria:

- at least 1 occurrences of a visit occurrence of Inpatient or ER visit (see Supplementary Table 5)

where event starts between all days Before and 1 days After index start date and event ends between 0 days Before and all days After index start date

Limit cohort of initial events to: all events per person.

Limit qualifying cohort to: all events per person.

End Date Strategy

Date Offset Exit Criteria
This cohort definition end date will be the index event's start date plus 7 days

Cohort Collapse Strategy:
Collapse cohort by era with a gap size of 180 days.

Acute renal failure events

Acute renal failure

Cohort Entry Events

People may enter the cohort when observing any of the following:

- condition occurrences of 'Acute Renal Failure' (see Supplementary Table 9).

Restrict entry events to having at least 1 visit occurrence of 'Inpatient or ER visit' (see Supplementary Table 5), starting anytime on or before cohort entry start date and ending between 0 days before and all days after cohort entry start date.

Cohort Exit

The cohort end date will be offset from index event's start date plus 30 days.

Cohort Eras

Entry events will be combined into cohort eras if they are within 30 days of each other.

Angioedema events

Angioedema condition record during an inpatient or ER visit; successive records with >7 day gap are considered independent episodes

Initial Event Cohort

People having any of the following:

- a condition occurrence of Angioedema (see Supplementary Table 10)

with continuous observation of at least 0 days prior and 0 days after event index date, and limit initial events to: all events per person.

For people matching the Primary Events, include:

Having any of the following criteria:

- at least 1 occurrences of a visit occurrence of Inpatient or ER visit (see Supplementary Table 5)

where event starts between all days Before and 0 days After index start date and event ends between 0 days Before and all days After index start date

Limit cohort of initial events to: all events per person.

Limit qualifying cohort to: all events per person.

End Date Strategy

Date Offset Exit Criteria
This cohort definition end date will be the index event's start date plus 7 days

Cohort Collapse Strategy:
Collapse cohort by era with a gap size of 30 days.

Cough events

Cough condition record of any type; successive records with > 90 day gap are considered independent episodes

Initial Event Cohort

People having any of the following:

- a condition occurrence of Cough (see Supplementary Table 11)

with continuous observation of at least 0 days prior and 0 days after event index date, and limit initial events to: all events per person.

Limit qualifying cohort to: all events per person.

End Date Strategy

Date Offset Exit Criteria
This cohort definition end date will be the index event's start date plus 1 days

Cohort Collapse Strategy:
Collapse cohort by era with a gap size of 90 days.

Gastrointestinal bleeding events

Cohort Entry Events

People may enter the cohort when observing any of the following:

- condition occurrences of 'Gastrointestinal hemorrhage GI bleeding' (see Supplementary Table 12).

Restrict entry events to having at least 1 visit occurrence of 'Inpatient or ER visit' (see Supplementary Table 5), starting anytime on or before cohort entry start date and ending between 0 days before and all days after cohort entry start date.

Cohort Exit

The cohort end date will be offset from index event's start date plus 7 days.

Cohort Eras

Entry events will be combined into cohort eras if they are within 30 days of each other.

Hyponatremia events

Cohort Entry Events

People enter the cohort when observing any of the following:

- condition occurrences of 'Hyponatremia' (see Supplementary Table 13).

Cohort Exit

The cohort end date will be offset from index event's start date plus 1 day.

Cohort Eras

Entry events will be combined into cohort eras if they are within 90 days of each other.

Hyperkalemia events

Condition record for hyperkalemia or potassium measurements > 5.6 mmol/L; successive records with >90 day gap are considered independent episodes

Initial Event Cohort

People having any of the following:

- a condition occurrence of Hyperkalemia (see Supplementary Table 14)
- a measurement of Potassium measurement (see Supplementary Table 15)
    - with value as number > 5.6
    - unit is any of: millimole per liter

with continuous observation of at least 0 days prior and 0 days after event index date, and limit initial events to: all events per person.

Limit qualifying cohort to: all events per person.

End Date Strategy

Date Offset Exit Criteria
This cohort definition end date will be the index event's start date plus 1 days

Cohort Collapse Strategy:
Collapse cohort by era with a gap size of 90 days.

Hypokalemia events

Hypokalemia condition record of any type; successive records with > 90 day gap are considered independent episodes

Initial Event Cohort

People having any of the following:

- a condition occurrence of Hypokalemia (see Supplementary Table 16)

with continuous observation of at least 0 days prior and 0 days after event index date, and limit initial events to: all events per person.

Limit qualifying cohort to: all events per person.

End Date Strategy

Date Offset Exit Criteria
This cohort definition end date will be the index event's start date plus 1 days

Cohort Collapse Strategy:
Collapse cohort by era with a gap size of 90 days.

Hypotension events

Hypotension condition record of any type; successive records with > 90 day gap are considered independent episodes

Initial Event Cohort

People having any of the following:

- a condition occurrence of Hypotension (see Supplementary Table 17)

with continuous observation of at least 0 days prior and 0 days after event index date, and limit initial events to: all events per person.

Limit qualifying cohort to: all events per person.

End Date Strategy

Date Offset Exit Criteria

This cohort definition end date will be the index event's start date plus 1 days

Cohort Collapse Strategy:

Collapse cohort by era with a gap size of 90 days.

Kidney disease events

Cohort Entry Events

People may enter the cohort when observing any of the following:

- condition occurrence of 'Chronic kidney disease' (see Supplementary Table 18) for the first time in the person's history.

Limit cohort entry events to the earliest event per person.

Restrict entry events to with any of the following criteria:

- having at least 1 condition occurrence of 'Chronic kidney disease' (see Supplementary Table 18), starting 1 days after cohort entry start date.
- having at least 1 procedure occurrence of 'Dialysis' (see Supplementary Table 19), starting between 0 days before and all days after cohort entry start date.
- having at least 1 observation of 'Dialysis' (see Supplementary Table 19), starting between 0 days before and all days after cohort entry start date.

Cohort Exit

The person exits the cohort at the end of continuous observation.

Cohort Eras

Entry events will be combined into cohort eras if they are within 0 days of each other.

Section C. Negative control outcomes

The sensitivity analyses based on negative control outcomes have been thoroughly described in [1]. We have followed the exact same process. The list of negative control outcomes can be seen in the list below:

Contact dermatitis
Senile hyperkeratosis
Onychomycosis due to dermatophyte
Impacted cerumen
Tear film insufficiency
Tobacco dependence syndrome
Presbyopia
Ingrowing nail
Somatic dysfunction of lumbar region
Verruca vulgaris
Wrist joint pain
Vaginitis and vulvovaginitis
Cervical somatic dysfunction
Injury of knee
Sprain of ankle
Herpes zoster without complication
Calcaneal spur
Hammer toe
Chondromalacia of patella
Strain of rotator cuff capsule
Acquired hallux valgus
Human papilloma virus infection
Non-toxic multinodular goiter
Acute conjunctivitis
Acquired trigger finger
Acquired keratoderma
Anal and rectal polyp
Nicotine dependence
Derangement of knee
Contusion of knee
Irregular periods
Feces contents abnormal
Regular astigmatism
Leukorrhea
Impingement syndrome of shoulder region
Epidermoid cyst

Melena
Absence of breast
Nonspecific tuberculin test reaction
Macular drusen
Crohn's disease
Endometriosis
Abnormal cervical smear
Changes in skin texture
Colostomy present
Psychalgia
Absent kidney
High risk sexual behavior
Difficulty sleeping
Opioid abuse
Cannabis abuse
Abrasion and/or friction burn of trunk without infection
Acid reflux
Complication due to Crohn's disease
Genetic predisposition
Ileostomy present
Passing flatus
Ganglion cyst
Hereditary thrombophilia
Foreign body in orifice
Cocaine abuse
Problem related to lifestyle
Kwashiorkor
Post-viral fatigue syndrome
Ptotic breast
Noise effects on inner ear
Abnormal pupil
Homocystinuria
Amputated foot
Effects of hunger
Late effect of contusion
Late effect of motor vehicle accident
Disproportion of reconstructed breast
Burn of forearm
Splinter of face, without major open wound
Wristdrop

Section D. Database descriptions

IBM® MarketScan® Commercial Database (CCAE)

The IBM® MarketScan® Commercial Database (CCAE) includes health insurance claims across the continuum of care (e.g., inpatient, outpatient, outpatient pharmacy, carve-out behavioral healthcare) as well as enrollment data from large employers and health plans across the United States who provide private healthcare coverage for more than 157 million employees, their spouses, and dependents. This administrative claims database includes a variety of fee- for-service, preferred provider organizations, and capitated health plans.

IBM® MarketScan® Multi-State Medicaid Database (MDCD)

The IBM® MarketScan® Multi-State Medicaid Database (MDCD) reflects the healthcare service use of individuals covered by Medicaid programs in numerous geographically dispersed states. The database contains the pooled healthcare experience of Medicaid enrollees, covered under fee-for-service and managed care plans. It includes records of inpatient services, inpatient admissions, outpatient services, and prescription drug claims, as well as information on long-term care. Data on eligibility and service and provider type are also included. In addition to standard demographic variables such as age and gender, the database includes variables such as federal aid category (income based, disability, Temporary Assistance for Needy Families) and race.

IBM® MarketScan® Medicare Supplemental Database (MDCR)

The IBM® MarketScan® Medicare Supplemental Database (MDCR) represents the health services of approximately 10 million retirees in the United States with Medicare supplemental coverage through employer-sponsored plans. This database contains primarily fee-for-service plans and includes health insurance claims across the continuum of care (e.g., inpatient, outpatient and outpatient pharmacy).

SUPPLEMENTARY TABLES

Supplementary Table 1. Hypertensive disorder. Concepts used to define hypertensive disorder.

Concept Id	Concept Name	Domain	Vocabulary	Excluded	Descendants	Mapped
316866	Hypertensive disorder	Condition	SNOMED	NO	YES	NO

Supplementary Table 2. Hypertension drugs. Concepts used to define hypertension treatments.

Concept Id	Concept Name	Domain	Vocabulary	Excluded	Descendants	Mapped
40235485	azilsartan	Drug	RxNorm	NO	YES	NO
40226742	olmesartan	Drug	RxNorm	NO	YES	NO
1398937	Clonidine	Drug	RxNorm	NO	YES	NO
1395058	Chlorthalidone	Drug	RxNorm	NO	YES	NO
1386957	Labetalol	Drug	RxNorm	NO	YES	NO
1373928	Hydralazine	Drug	RxNorm	NO	YES	NO
1373225	Perindopril	Drug	RxNorm	NO	YES	NO
1367500	Losartan	Drug	RxNorm	NO	YES	NO
1363749	Fosinopril	Drug	RxNorm	NO	YES	NO
1363053	Doxazosin	Drug	RxNorm	NO	YES	NO
1353776	Felodipine	Drug	RxNorm	NO	YES	NO
1353766	Propranolol	Drug	RxNorm	NO	YES	NO
1351557	candesartan	Drug	RxNorm	NO	YES	NO
1350489	Prazosin	Drug	RxNorm	NO	YES	NO
1347384	irbesartan	Drug	RxNorm	NO	YES	NO
1346823	carvedilol	Drug	RxNorm	NO	YES	NO
1346686	eprosartan	Drug	RxNorm	NO	YES	NO
1345858	Pindolol	Drug	RxNorm	NO	YES	NO
1344965	Guanfacine	Drug	RxNorm	NO	YES	NO
1342439	trandolapril	Drug	RxNorm	NO	YES	NO
1341927	Enalapril	Drug	RxNorm	NO	YES	NO
1341238	Terazosin	Drug	RxNorm	NO	YES	NO
1340128	Captopril	Drug	RxNorm	NO	YES	NO
1338005	Bisoprolol	Drug	RxNorm	NO	YES	NO
1335471	benazepril	Drug	RxNorm	NO	YES	NO
1334456	Ramipril	Drug	RxNorm	NO	YES	NO
1332418	Amlodipine	Drug	RxNorm	NO	YES	NO
1331235	quinapril	Drug	RxNorm	NO	YES	NO
1328165	Diltiazem	Drug	RxNorm	NO	YES	NO
1327978	Penbutolol	Drug	RxNorm	NO	YES	NO
1326012	Isradipine	Drug	RxNorm	NO	YES	NO
1322081	Betaxolol	Drug	RxNorm	NO	YES	NO
1319998	Acebutolol	Drug	RxNorm	NO	YES	NO
1319880	Nisoldipine	Drug	RxNorm	NO	YES	NO
1318853	Nifedipine	Drug	RxNorm	NO	YES	NO
1318137	Nicardipine	Drug	RxNorm	NO	YES	NO
1317967	aliskiren	Drug	RxNorm	NO	YES	NO
1317640	telmisartan	Drug	RxNorm	NO	YES	NO
1314577	nebivolol	Drug	RxNorm	NO	YES	NO
1314002	Atenolol	Drug	RxNorm	NO	YES	NO
1313200	Nadolol	Drug	RxNorm	NO	YES	NO
1310756	moexipril	Drug	RxNorm	NO	YES	NO

1309799	eplerenone	Drug	RxNorm	NO	YES	NO
1309068	Minoxidil	Drug	RxNorm	NO	YES	NO
1308842	valsartan	Drug	RxNorm	NO	YES	NO
1308216	Lisinopril	Drug	RxNorm	NO	YES	NO
1307863	Verapamil	Drug	RxNorm	NO	YES	NO
1307046	Metoprolol	Drug	RxNorm	NO	YES	NO
1305447	Methyldopa	Drug	RxNorm	NO	YES	NO
991382	Amiloride	Drug	RxNorm	NO	YES	NO
978555	Indapamide	Drug	RxNorm	NO	YES	NO
974166	Hydrochlorothiazide	Drug	RxNorm	NO	YES	NO
970250	Spironolactone	Drug	RxNorm	NO	YES	NO
956874	Furosemide	Drug	RxNorm	NO	YES	NO
942350	torsemide	Drug	RxNorm	NO	YES	NO
932745	Bumetanide	Drug	RxNorm	NO	YES	NO
907013	Metolazone	Drug	RxNorm	NO	YES	NO
904542	Triamterene	Drug	RxNorm	NO	YES	NO

Supplementary Table 3. Thiazide or thiazide-like diuretics. Concepts used to define exposure to thiazide or thiazide-like diuretics.

Concept Id	Concept Name	Domain	Vocabulary	Excluded	Descendants	Mapped
1395058	Chlorthalidone	Drug	RxNorm	NO	YES	NO
974166	Hydrochlorothiazide	Drug	RxNorm	NO	YES	NO
978555	Indapamide	Drug	RxNorm	NO	YES	NO
907013	Metolazone	Drug	RxNorm	NO	YES	NO

Supplementary Table 4. ACE inhibitors. Concepts used to define exposure to ACE inhibitors.

Concept Id	Concept Name	Domain	Vocabulary	Excluded	Descendants	Mapped
1308216	Lisinopril	Drug	RxNorm	NO	YES	NO
1310756	moexipril	Drug	RxNorm	NO	YES	NO
1331235	quinapril	Drug	RxNorm	NO	YES	NO
1334456	Ramipril	Drug	RxNorm	NO	YES	NO
1335471	benazepril	Drug	RxNorm	NO	YES	NO
1340128	Captopril	Drug	RxNorm	NO	YES	NO
1341927	Enalapril	Drug	RxNorm	NO	YES	NO
1342439	trandolapril	Drug	RxNorm	NO	YES	NO
1363749	Fosinopril	Drug	RxNorm	NO	YES	NO
1373225	Perindopril	Drug	RxNorm	NO	YES	NO

Supplementary Table 5. Inpatient or ER visit. Concepts used to define inpatient or ER visit.

Concept Id	Concept Name	Domain	Vocabulary	Excluded	Descendants	Mapped
262	Emergency Room and Inpatient Visit	Visit	Visit	NO	YES	NO
9201	Inpatient Visit	Visit	Visit	NO	YES	NO
9203	Emergency Room Visit	Visit	Visit	NO	YES	NO

Supplementary Table 6. Acute myocardial Infarction. Concepts used to define acute myocardial infarction.

Concept Id	Concept Name	Domain	Vocabulary	Excluded	Descendants	Mapped
314666	Old myocardial infarction	Condition	SNOMED	YES	YES	NO
4329847	Myocardial infarction	Condition	SNOMED	NO	YES	NO

Supplementary Table 7. Heart Failure. Concepts used to define heart failure.

Concept Id	Concept Name	Domain	Vocabulary	Excluded	Descendants	Mapped
315295	Congestive rheumatic heart failure	Condition	SNOMED	YES	YES	NO
316139	Heart failure	Condition	SNOMED	NO	YES	NO

Supplementary Table 8. Stroke (ischemic or hemorrhagic). Concepts used to define stroke (ischemic or hemorrhagic).

Concept Id	Concept Name	Domain	Vocabulary	Excluded	Descendants	Mapped
372924	Cerebral artery occlusion	Condition	SNOMED	NO	NO	NO
375557	Cerebral embolism	Condition	SNOMED	NO	NO	NO
376713	Cerebral hemorrhage	Condition	SNOMED	NO	NO	NO
432923	Subarachnoid hemorrhage	Condition	SNOMED	NO	NO	NO
439847	Intracranial hemorrhage	Condition	SNOMED	NO	NO	NO
441874	Cerebral thrombosis	Condition	SNOMED	NO	NO	NO
443454	Cerebral infarction	Condition	SNOMED	NO	YES	NO

Supplementary Table 9. Acute renal failure. Concepts used to define acute renal failure.

Concept Id	Concept Name	Domain	Vocabulary	Excluded	Descendants	Mapped
197320	Acute renal failure syndrome	Condition	SNOMED	NO	YES	NO
432961	Acute renal papillary necrosis with renal failure	Condition	SNOMED	NO	YES	NO
444044	Acute tubular necrosis	Condition	SNOMED	NO	YES	NO

Supplementary Table 10. Angioedema. Concepts used to define angioedema across all databases.

Concept Id	Concept Name	Domain	Vocabulary	Excluded	Descendants	Mapped
432791	Angioedema	Condition	SNOMED	NO	YES	NO

Supplementary Table 11. Cough. Concepts used to define cough across all databases.

Concept Id	Concept Name	Domain	Vocabulary	Excluded	Descendants	Mapped
254761	Cough	Condition	SNOMED	NO	YES	NO

Supplementary Table 12. Gastrointestinal hemorrhage GI bleeding. Concepts used to define gastrointestinal bleeding across all databases.

Concept Id	Concept Name	Domain	Vocabulary	Excluded	Descendants	Mapped
23808	Chronic peptic ulcer without hemorrhage, without perforation AND without obstruction	Condition	SNOMED	YES	YES	NO
24973	Chronic peptic ulcer without hemorrhage AND without perforation but with obstruction	Condition	SNOMED	YES	YES	NO
28779	Bleeding esophageal varices	Condition	SNOMED	NO	YES	NO
192671	Gastrointestinal hemorrhage	Condition	SNOMED	NO	YES	NO
195584	Acute peptic ulcer without hemorrhage AND without perforation but with obstruction	Condition	SNOMED	YES	YES	NO
197925	Hemorrhage of rectum and anus	Condition	SNOMED	YES	YES	NO
198798	Dieulafoy's vascular malformation	Condition	SNOMED	NO	YES	NO
200769	Chronic gastric ulcer without hemorrhage, without perforation AND without obstruction	Condition	SNOMED	YES	YES	NO
434400	Chronic gastrojejunal ulcer without hemorrhage AND without perforation but with obstruction	Condition	SNOMED	YES	YES	NO
438795	Chronic gastrojejunal ulcer without hemorrhage, without perforation AND without obstruction	Condition	SNOMED	YES	YES	NO
443530	Hematochezia	Condition	SNOMED	YES	YES	NO
2002608	Control of hemorrhage and suture of ulcer of stomach or duodenum	Procedure	ICD9Proc	NO	YES	NO
2108878	Esophagoscopy, flexible, transoral; with control of bleeding, any method	Procedure	CPT4	NO	YES	NO
2108900	Esophagogastroduodenoscopy, flexible, transoral; with control of bleeding, any method	Procedure	CPT4	NO	YES	NO
4027663	Peptic ulcer	Condition	SNOMED	NO	YES	NO

4101104	Gastrojejunal ulcer without hemorrhage AND without perforation	Condition	SNOMED	YES	YES	NO
4112183	Esophageal varices with bleeding, associated with another disorder	Condition	SNOMED	NO	YES	NO
4138962	Acute duodenal ulcer without hemorrhage AND without perforation	Condition	SNOMED	YES	YES	NO
4147683	Acute gastrojejunal ulcer without hemorrhage AND without perforation	Condition	SNOMED	NO	NO	NO
4163865	Acute peptic ulcer without hemorrhage AND without perforation	Condition	SNOMED	YES	YES	NO
4177387	Chronic gastrojejunal ulcer without hemorrhage AND without perforation	Condition	SNOMED	YES	YES	NO
4195231	Acute gastric ulcer without hemorrhage AND without perforation	Condition	SNOMED	YES	YES	NO
4198381	Duodenal ulcer disease	Condition	SNOMED	NO	YES	NO
4204555	Chronic peptic ulcer without hemorrhage AND without perforation	Condition	SNOMED	YES	YES	NO
4209746	Duodenal ulcer without hemorrhage AND without perforation	Condition	SNOMED	YES	YES	NO
4222896	Chronic duodenal ulcer without hemorrhage AND without perforation	Condition	SNOMED	YES	YES	NO
4248429	Gastric ulcer without hemorrhage AND without perforation	Condition	SNOMED	YES	YES	NO
4265600	Gastric ulcer	Condition	SNOMED	NO	YES	NO
4291028	Peptic ulcer without hemorrhage AND without perforation	Condition	SNOMED	YES	YES	NO
4296611	Chronic gastric ulcer without hemorrhage AND without perforation	Condition	SNOMED	YES	YES	NO
40482685	Angiodysplasia of duodenum	Condition	SNOMED	NO	YES	NO

Supplementary Table 13. Hyponatremia. Concepts used to define hyponatremia across all databases.

Concept Id	Concept Name	Domain	Vocabulary	Excluded	Descendants	Mapped
435515	Hypo-osmolality and or hyponatremia	Condition	SNOMED	NO	YES	NO
4232311	Hyponatremia	Condition	SNOMED	NO	YES	NO

Supplementary Table 14. Hyperkalemia. Concepts used to define hyperkalemia across all databases.

Concept Id	Concept Name	Domain	Vocabulary	Excluded	Descendants	Mapped
434610	Hyperkalemia	Condition	SNOMED	NO	YES	NO

Supplementary Table 15. Potassium measurement. Concepts used to define potassium measurements across all databases.

Concept Id	Concept Name	Domain	Vocabulary	Excluded	Descendants	Mapped
4245152	Potassium measurement	Measurement	SNOMED	NO	YES	NO
4276440	Potassium level - finding	Condition	SNOMED	NO	YES	NO
40789893	Potassium | Bld-Ser-Plas	Measurement	LOINC	NO	YES	NO

Supplementary Table 16. Hypokalemia. Concepts used to define hypokalemia across all databases.

Concept Id	Concept Name	Domain	Vocabulary	Excluded	Descendants	Mapped
437833	Hypokalemia	Condition	SNOMED	NO	YES	NO
45769152	Bartter syndrome	Condition	SNOMED	YES	YES	NO

Supplementary Table 17. Hypotension. Concepts used to define hypotension across all databases.

Concept Id	Concept Name	Domain	Vocabulary	Excluded	Descendants	Mapped
313232	Hemodialysis-associated hypotension	Observation	SNOMED	YES	YES	NO
314432	Maternal hypotension syndrome	Condition	SNOMED	YES	YES	NO
317002	Low blood pressure	Condition	SNOMED	NO	YES	NO

Supplementary Table 18. Chronic kidney disease. Concepts used to define chronic kidney disease across all databases.

Concept Id	Concept Name	Domain	Vocabulary	Excluded	Descendants	Mapped
192279	Diabetic renal disease	Condition	SNOMED	NO	YES	NO
192359	Renal failure syndrome	Condition	SNOMED	NO	YES	NO
193016	Cystic disease of kidney	Condition	SNOMED	NO	YES	NO
193253	Nephritis	Condition	SNOMED	NO	NO	NO
194385	Aneurysm of renal artery	Condition	SNOMED	NO	YES	NO
195014	Renal failure following molar AND/OR ectopic pregnancy	Condition	SNOMED	YES	YES	NO
195289	Goodpasture's syndrome	Condition	SNOMED	YES	YES	NO
195314	Nephrotic syndrome	Condition	SNOMED	NO	YES	NO
195737	Hemorrhagic nephroso-nephritis	Condition	SNOMED	YES	YES	NO
195834	Atherosclerosis of renal artery	Condition	SNOMED	NO	YES	NO
197930	Renal hypertension complicating pregnancy, childbirth and the puerperium	Condition	SNOMED	YES	YES	NO
201313	Hypertensive renal disease	Condition	SNOMED	NO	YES	NO
261071	Glomerulosclerosis	Condition	SNOMED	NO	YES	NO
444044	Acute tubular necrosis	Condition	SNOMED	NO	YES	NO
4066005	Post-delivery acute renal failure with postnatal problem	Condition	SNOMED	YES	YES	NO
4103224	Interstitial nephritis	Condition	SNOMED	NO	YES	NO
4128219	Urate nephropathy	Condition	SNOMED	NO	YES	NO
4263367	Glomerulonephritis	Condition	SNOMED	NO	YES	NO
37116834	Postpartum acute renal failure	Condition	SNOMED	YES	YES	NO
43530912	Induced termination of pregnancy	Condition	SNOMED	YES	YES	NO

	complicated by renal failure					
45769152	Bartter syndrome	Condition	SNOMED	YES	YES	NO
46271022	Chronic kidney disease	Condition	SNOMED	NO	YES	NO

Supplementary Table 19. Dialysis. Concepts used to define dialysis across all databases.

Concept Id	Concept Name	Domain	Vocabulary	Excluded	Descendants	Mapped
4032243	Dialysis procedure	Procedure	SNOMED	NO	YES	NO
4090651	Dialysis finding	Observation	SNOMED	NO	YES	NO
45889365	Dialysis Services and Procedures	Procedure	CPT4	NO	YES	NO

Supplementary Table 20: Baseline characteristics of patients in CCAE. THZ: new users of thiazide or thiazide-like diuretics. ACE: new users of ACE inhibitors.

	Before stratification			After stratification		
Characteristic	THZ	ACE	Std. diff	THZ	ACE	Std. diff
Age in years	47.7	49	-0.12	48.7	48.7	0
Sex: female	60.6%	38.1%	0.46	44.5%	44.2%	0.01
Medical history: General						
Attention deficit hyperactivity disorder	0.6%	0.6%	0	0.6%	0.6%	0
Chronic obstructive lung disease	1.3%	1.5%	-0.02	1.5%	1.4%	0.01
Crohn's disease	0.2%	0.2%	0	0.2%	0.2%	0
Depressive disorder	3.8%	3.3%	0.03	3.6%	3.5%	0.01
Diabetes mellitus	0%	0.2%	-0.05	0.1%	0.2%	-0.03
Gastroesophageal reflux disease	5.4%	5.3%	0	5.5%	5.3%	0.01
Gastrointestinal hemorrhage	0.4%	0.4%	0	0.4%	0.4%	0
Human immunodeficiency virus infection	0.3%	0.2%	0.01	0.2%	0.2%	0
Hyperlipidemia	16.6%	23.3%	-0.17	21.1%	21.5%	-0.01
Hypertensive disorder	0.2%	0.3%	-0.01	0.3%	0.3%	0
Obesity	8.8%	7.7%	0.04	8%	7.9%	0
Osteoarthritis	1.8%	1.8%	0	1.9%	1.8%	0.01
Pneumonia	0.3%	0.3%	-0.01	0.4%	0.3%	0.01
Psoriasis	0.8%	0.9%	-0.01	0.9%	0.8%	0
Rheumatoid arthritis	0.8%	0.7%	0.01	0.8%	0.7%	0.01
Schizophrenia	0%	0%	0	0%	0%	0
Ulcerative colitis	0.2%	0.2%	-0.01	0.2%	0.2%	0
Urinary tract infectious disease	6%	4.7%	0.06	5.3%	5%	0.01
Viral hepatitis C	0.1%	0.2%	-0.01	0.1%	0.1%	0
Medical history: Cardiovascular disease						
Atrial fibrillation	0.2%	0.4%	-0.03	0.3%	0.3%	0
Cerebrovascular disease	0.2%	0.4%	-0.04	0.4%	0.4%	0
Coronary arteriosclerosis	0.5%	1.2%	-0.08	0.8%	0.9%	-0.01
Heart disease	0.2%	0.2%	-0.02	0.2%	0.2%	0
Heart failure	0.1%	0.1%	-0.01	0.1%	0.1%	0
Ischemic heart disease	0%	0%	-0.01	0%	0%	0
Pulmonary embolism	0.1%	0.1%	0	0.1%	0.1%	0
Venous thrombosis	0.1%	0.1%	0	0.1%	0.1%	0
Medical history: Neoplasms						
Malignant lymphoma	0.1%	0.1%	0	0.1%	0.1%	0.01
Malignant neoplastic disease	0.1%	0.1%	0	0.1%	0.1%	0

Primary malignant neoplasm of prostate	0.3%	0.5%	-0.03	0.5%	0.5%	0

Table 21: Baseline characteristics of patients in MDCD. THZ: new users of thiazide or thiazide-like diuretics. ACE: new users of ACE inhibitors.

	Before stratification			After stratification		
Characteristic	THZ	ACE	Std. diff	THZ	ACE	Std. diff
Age in years	42.4	45.4	-0.2	44.6	44.4	0.01
Sex: female	70.3%	52.2%	0.38	58.6%	58.5%	0
Medical history: General						
Attention deficit hyperactivity disorder	1.6%	2.1%	-0.03	2.1%	1.9%	0.01
Chronic obstructive lung disease	7.2%	10.7%	-0.12	10.1%	9.4%	0.03
Crohn's disease	0.3%	0.3%	-0.01	0.3%	0.3%	0
Depressive disorder	10.1%	10.8%	-0.02	10.9%	10.6%	0.01
Diabetes mellitus	0.1%	0.6%	-0.09	0.2%	0.4%	-0.04
Gastroesophageal reflux disease	8.7%	9.3%	-0.02	9.4%	9.1%	0.01
Gastrointestinal hemorrhage	0.7%	1%	-0.03	0.9%	0.9%	0
Human immunodeficiency virus infection	1.3%	1%	0.03	1.1%	1.1%	0
Hyperlipidemia	14.4%	24.2%	-0.25	20.6%	20.8%	-0.01
Obesity	19.7%	16.6%	0.08	17.8%	17.6%	0.01
Osteoarthritis	5.2%	6.2%	-0.04	6.1%	5.8%	0.01
Pneumonia	1.4%	1.8%	-0.04	1.7%	1.6%	0
Psoriasis	0.6%	0.8%	-0.02	0.8%	0.7%	0.01
Rheumatoid arthritis	1.2%	1.3%	-0.01	1.3%	1.3%	0
Schizophrenia	3%	3.3%	-0.02	3.3%	3.2%	0
Ulcerative colitis	0.1%	0.2%	-0.01	0.2%	0.2%	0
Urinary tract infectious disease	11.2%	10%	0.04	10.5%	10.2%	0.01
Viral hepatitis C	1.6%	1.9%	-0.02	1.8%	1.8%	0
Medical history: Cardiovascular disease						
Atrial fibrillation	0.5%	1%	-0.05	0.9%	0.8%	0.01
Cerebrovascular disease	0.6%	1.1%	-0.06	0.9%	0.9%	0
Coronary arteriosclerosis	0.7%	1.9%	-0.1	1.4%	1.3%	0.01
Heart disease	0.4%	0.8%	-0.05	0.6%	0.6%	0
Heart failure	0.5%	0.8%	-0.04	0.8%	0.7%	0.01
Ischemic heart disease	0%	0%	-0.02	0%	0%	0
Pulmonary embolism	0.3%	0.3%	0	0.3%	0.3%	0.01
Venous thrombosis	0.2%	0.2%	0.01	0.2%	0.2%	0.01
Medical history: Neoplasms						
Malignant lymphoma	0.1%	0.1%	-0.01	0.1%	0.1%	0.01
Malignant neoplastic disease	0.1%	0.2%	-0.01	0.1%	0.2%	0
Primary malignant neoplasm of prostate	0.2%	0.4%	-0.03	0.3%	0.3%	0

Supplementary Table 22: Baseline characteristics of patients in MDCR. THZ: new users of thiazide or thiazide-like diuretics. ACE: new users of ACE inhibitors.

	Before stratification			After stratification		
Characteristic	THZ	ACE	Std. diff	THZ	ACE	Std. diff
Age in years	74.4	74	0.05	74.3	74.1	0.03
Sex: female	64.6%	52%	0.26	55.1%	55.6%	-0.01
Medical history: General						
Attention deficit hyperactivity disorder	0%	0.1%	-0.01	0%	0.1%	-0.01
Chronic obstructive lung disease	6.4%	6.9%	-0.02	6.9%	6.7%	0.01
Crohn's disease	0.1%	0.2%	-0.01	0.2%	0.2%	0
Depressive disorder	2.7%	3%	-0.02	2.9%	2.9%	0
Diabetes mellitus	0%	0%	-0.02	0%	0%	-0.01
Gastroesophageal reflux disease	7.4%	7.5%	0	7.7%	7.4%	0.01
Gastrointestinal hemorrhage	1%	1.3%	-0.02	1.2%	1.2%	0
Human immunodeficiency virus infection	0%	0.1%	-0.02	0%	0.1%	-0.01
Hyperlipidemia	22.8%	28.8%	-0.14	27%	26.8%	0
Obesity	2.7%	3%	-0.02	2.9%	2.8%	0
Osteoarthritis	6.4%	5.9%	0.02	6.2%	5.9%	0.01
Pneumonia	0.3%	0.4%	-0.02	0.3%	0.3%	0
Psoriasis	0.8%	0.9%	0	0.8%	0.9%	-0.01
Rheumatoid arthritis	1.6%	1.5%	0.01	1.5%	1.5%	0
Schizophrenia	0.1%	0.1%	-0.01	0.1%	0.1%	0
Ulcerative colitis	0.3%	0.3%	-0.01	0.3%	0.3%	0
Urinary tract infectious disease	7.4%	8.4%	-0.04	8.3%	8%	0.01
Viral hepatitis C	0.1%	0.1%	-0.01	0.1%	0.1%	0
Medical history: Cardiovascular disease						
Atrial fibrillation	2.5%	3.2%	-0.04	3.1%	2.9%	0.02
Cerebrovascular disease	1.5%	2.7%	-0.08	2.4%	2.4%	0
Coronary arteriosclerosis	3%	5.4%	-0.12	4.6%	4.4%	0.01
Heart disease	0.4%	0.7%	-0.04	0.6%	0.6%	0
Heart failure	0.2%	0.3%	-0.02	0.2%	0.2%	0
Ischemic heart disease	0.1%	0.1%	-0.02	0.1%	0.1%	-0.01
Pulmonary embolism	0.1%	0.1%	-0.01	0.1%	0.1%	0
Venous thrombosis	0.3%	0.3%	0	0.3%	0.3%	0
Medical history: Neoplasms						
Malignant lymphoma	0.4%	0.4%	0	0.4%	0.4%	0
Malignant neoplastic disease	0.2%	0.3%	-0.01	0.3%	0.3%	0

Primary malignant neoplasm of prostate	3.1%	3.9%	-0.04	3.9%	3.7%	0.01

Supplementary Table 23. Risk group sizes. Number of patients, person years, and events in risk strata of acute MI across all databases. RG-1 represents patients at acute MI risk below 1%, RG-2 represents patients at acute Mi risk between 1% and 1.5%, and RG-3 represents patients at acute MI risk larger than 1.5%.

		Thiazide or thiazide-like diuretics			ACE inhibitors		
Database	Risk group	Patients	Person years	Outcomes	Patients	Person years	Outcomes
CCAE	RG-1	347,892	200,792	368	874,820	550,857	1,500
	RG-2	5,576	2,760	23	37,950	23,408	169
	RG-3	2,358	1,042	14	17,599	9,902	144
MDCD	RG-1	39,144	14,584	22	61,229	28,408	109
	RG-2	7,798	3,371	13	19,066	9,823	78
	RG-3	7,893	3,484	41	26,197	13,250	253
MDCR	RG-1	9,635	6,861	19	22,407	16,157	55
	RG-2	14,944	9,985	48	40,296	29,365	216
	RG-3	13,303	7,796	94	43,149	29,468	461

Supplementary Table 24. Prediction populations. Sample sizes of several groups of interest in the development and (internal validation) of the derived acute MI risk prediction models in each database. Entire population refers to the sample size of the entire population of combined patients receiving thiazide or thiazide-like diuretics (treatment) and ACE inhibitors (comparator). Matched population refers to the propensity score matched population (1:1 propensity score matching) on which the acute MI prediction model was actually developed in each database. Treatment population refers to the population subset treated with thiazide or thiazide-like diuretics, while the comparator population refers to the population subset treated with ACE inhibitors.

Population	CCAE	MDCD	MDCR
Entire population	1,286,195	161,327	143,734
Matched	674,648 (52.5%)	87,676 (54.3%)	71,808 (50.0%)
Treatment	355,826 (27.7%)	54,835 (34.0%)	37,882 (26.4%)
Comparator	930,369 (72.3%)	106,492 (66.0%)	105,852 (73.6%)

Supplementary Table 25. Variable importance in CCAE. Covariates with the 20 largest coefficients included in the acute MI risk prediction model in database CCAE.

Covariate	Coefficient
age in years	3.782485
gender = MALE	0.747513
condition occurrence during day -365 through 0 days relative to index: Nicotine dependence	0.637835
condition occurrence during day -365 through 0 days relative to index: Coronary arteriosclerosis	0.548153
condition occurrence during day -30 through 0 days relative to index: Chronic obstructive lung disease	0.510603
condition occurrence during day -365 through 0 days relative to index: Coronary atherosclerosis	0.447896
condition occurrence during day -365 through 0 days relative to index: Type 2 diabetes mellitus without complication	0.348854
condition occurrence during day -365 through 0 days relative to index: Tobacco dependence syndrome	0.339254
condition occurrence during day -365 through 0 days relative to index: Disorder of muscle	0.339192
condition occurrence during day -365 through 0 days relative to index: Disorder of body system	0.3372
drug exposure during day -365 through 0 days relative to index: acetaminophen 500 MG / hydrocodone bitartrate 10 MG Oral Tablet	0.285335
condition occurrence during day -365 through 0 days relative to index: Dyspnea	0.277757
drug era during day -365 through 0 days relative to index: varenicline	0.265711
condition occurrence during day -365 through 0 days relative to index: Nausea and vomiting	0.262168
drug era during day -365 through 0 days relative to index: paroxetine	0.255913
drug era during day -365 through 0 days relative to index: triamcinolone	-0.22833
drug era during day -30 through 0 days relative to index: albuterol	0.22608
drug era during day -30 through 0 days relative to index: azithromycin	0.215757
drug era during day -30 through 0 days relative to index: methylprednisolone	0.209836
condition occurrence during day -365 through 0 days relative to index: Senile hyperkeratosis	-0.2084

Supplementary Table 26. Variable importance in MDCD. Covariates with the 20 largest coefficients included in the acute MI risk prediction model in database MDCD.

Covariate	Coefficient
age in years	2.763943
condition occurrence during day -365 through 0 days relative to index: Essential hypertension	-0.89741
condition occurrence during day -365 through 0 days relative to index: Viral hepatitis C	0.530233
condition occurrence during day -365 through 0 days relative to index: Chronic obstructive lung disease	0.45505
condition occurrence during day -365 through 0 days relative to index: Conjunctivitis	0.450288
condition occurrence during day -365 through 0 days relative to index: Benign essential hypertension	-0.42922
condition occurrence during day -365 through 0 days relative to index: Type 2 diabetes mellitus	0.39883
gender = MALE	0.39651
condition occurrence during day -30 through 0 days relative to index: Benign essential hypertension	-0.37672
drug exposure during day -30 through 0 days relative to index: gabapentin 800 MG Oral Tablet	0.334705
condition occurrence during day -365 through 0 days relative to index: Chest pain	0.321369
condition occurrence during day -365 through 0 days relative to index: Atherosclerosis of coronary artery without angina pectoris	0.309086
condition occurrence during day -365 through 0 days relative to index: Vitamin D deficiency	-0.30105
drug exposure during day -365 through 0 days relative to index: acetaminophen 325 MG / oxycodone hydrochloride 10 MG Oral Tablet	-0.26092
drug exposure during day -365 through 0 days relative to index: acetaminophen 325 MG / hydrocodone bitartrate 10 MG Oral Tablet	0.248342
condition occurrence during day -365 through 0 days relative to index: Nicotine dependence	0.246476
condition occurrence during day -365 through 0 days relative to index: Tobacco dependence syndrome	0.244977
drug era during day -30 through 0 days relative to index: ibuprofen	-0.24306
condition occurrence during day -365 through 0 days relative to index: Dyspnea	0.242227
drug era during day -30 through 0 days relative to index: baclofen	0.236925

Supplementary Table 27. Variable importance in MDCR. Covariates with the 20 largest coefficients included in the acute MI risk prediction model in database MDCR.

Covariate	Coefficient
age in years	2.734837
drug exposure during day -365 through 0 days relative to index: levothyroxine sodium 0.112 MG Oral Tablet	0.638691
gender = MALE	0.423371
drug era during day -365 through 0 days relative to index: donepezil	0.368718
condition occurrence during day -365 through 0 days relative to index: Right upper quadrant pain	0.332841
drug era during day -365 through 0 days relative to index: morphine	0.319726
condition occurrence during day -365 through 0 days relative to index: Coronary arteriosclerosis	0.303143
condition occurrence during day -365 through 0 days relative to index: Gastritis	0.285354
condition occurrence during day -365 through 0 days relative to index: Congestive heart failure	0.285295
condition occurrence during day -30 through 0 days relative to index: Osteoarthritis of knee	0.284456
drug era during day -365 through 0 days relative to index: chlorhexidine	0.274925
condition occurrence during day -365 through 0 days relative to index: Inflammatory dermatosis	-0.26152
drug era during day -365 through 0 days relative to index: erythromycin	0.256304
drug exposure during day -365 through 0 days relative to index: acetaminophen 650 MG / propoxyphene napsylate 100 MG Oral Tablet	0.240047
drug era during day -365 through 0 days relative to index: celecoxib	0.239481
condition occurrence during day -365 through 0 days relative to index: Disorder of carotid artery	0.233565
condition occurrence during day -365 through 0 days relative to index: Hyperlipidemia	-0.22439
drug exposure during day -365 through 0 days relative to index: tramadol hydrochloride 50 MG Oral Tablet	0.21753
drug era during day -30 through 0 days relative to index: albuterol	0.202049
condition occurrence during day -365 through 0 days relative to index: Diverticular disease of colon	-0.1937

SUPLLEMENTARY FIGURES

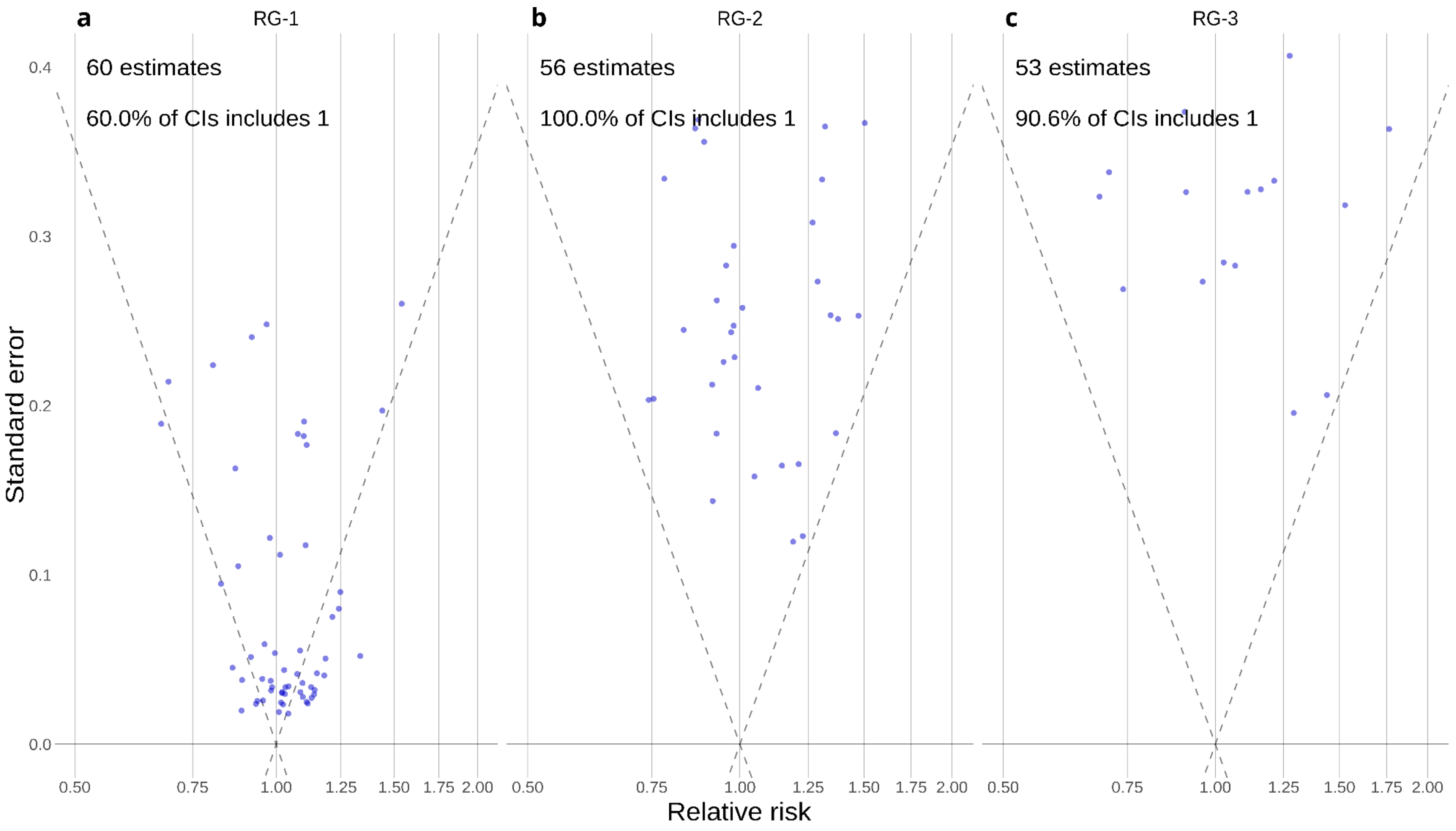

Supplementary Figure 1. Systematic error in risk groups of CCAE. Effect size estimates for the negative controls (true hazard ratio = 1) within strata of predicted acute MI risk. Estimates below the diagonal dashed lines are statistically significant (different from the true effect size, alpha = 0.05). RG-1 represents patients in CCAE whose acute MI predicted risk is below 1% (a); RG-2 represents patients whose acute MI predicted risk is between 1% and 1.5% (b); RG-3 represents patients whose acute MI predicted risk is larger than 1.5% (c). A well-calibrated estimator should have the true effect size (HR = 1) within the 95 percent confidence interval 95 percent of times.

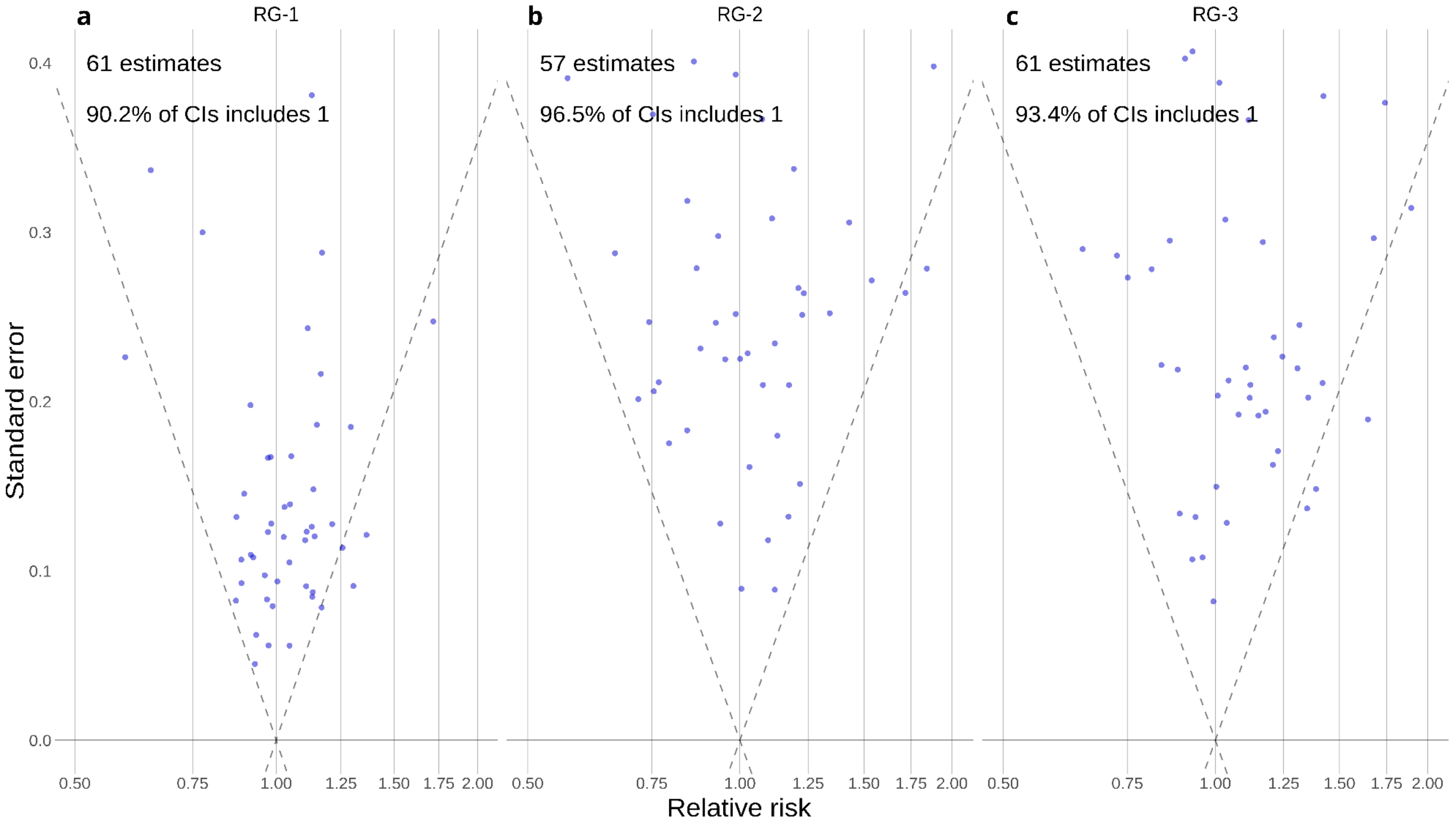

Supplementary Figure 2. Systematic error in risk groups of MDCD. Effect size estimates for the negative controls (true hazard ratio = 1) within strata of predicted acute MI risk in MDCD. Estimates below the diagonal dashed lines are statistically significant (different from the true effect size, alpha = 0.05). RG-1 represents patients in MDCD whose acute MI predicted risk is below 1% (a); RG-2 represents patients whose acute MI predicted risk is between 1% and 1.5% (b); RG-3 represents patients whose acute MI predicted risk is larger than 1.5% (c). A well-calibrated estimator should have the true effect size within the 95 percent confidence interval 95 percent of times.

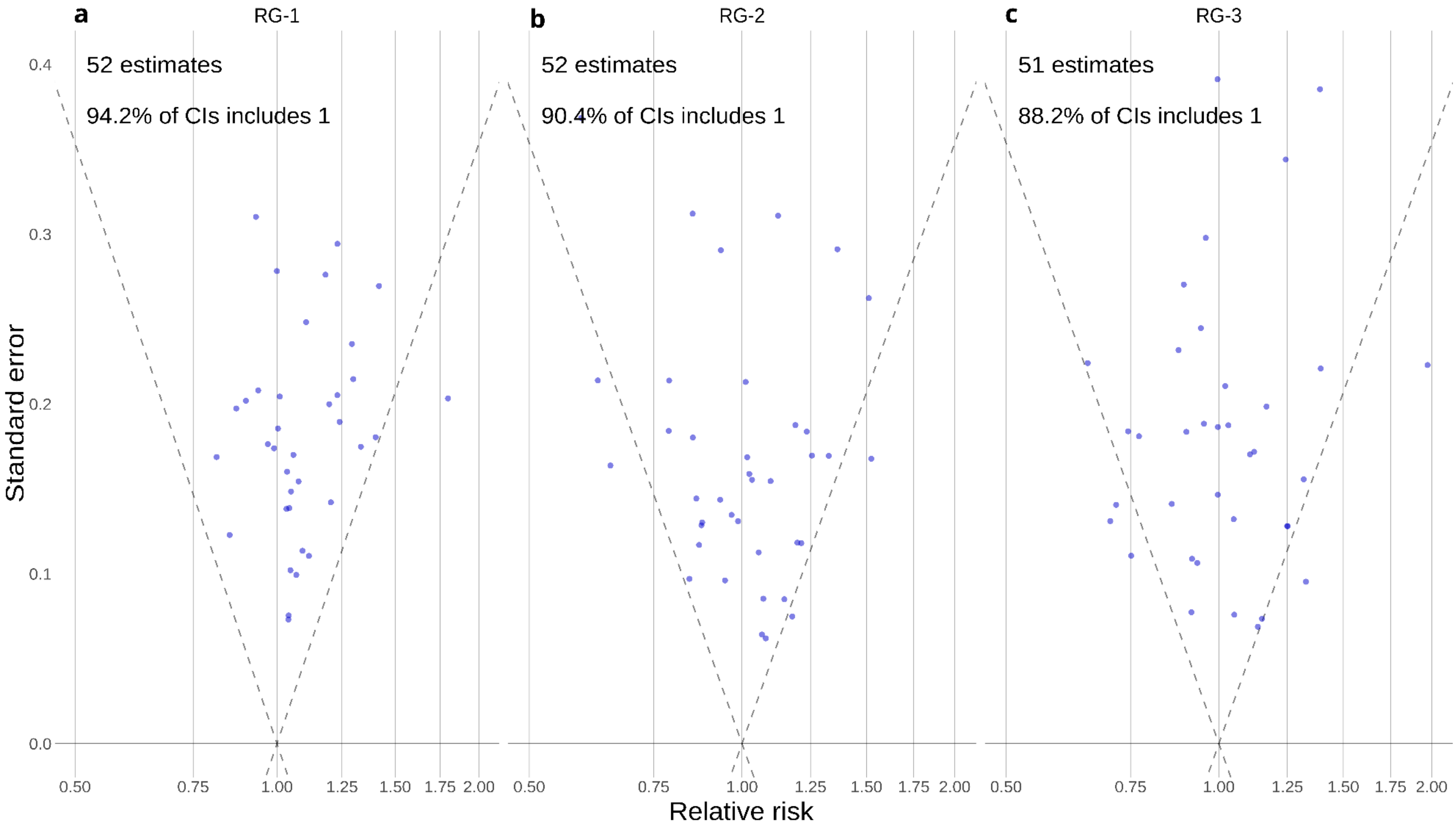

Supplementary Figure 3. Systematic error in risk groups of MDCR. Effect size estimates for the negative controls (true hazard ratio = 1) within strata of predicted acute MI risk in MDCR. Estimates below the diagonal dashed lines are statistically significant (different from the true effect size, alpha = 0.05). RG-1 represents patients in MDCR whose acute MI predicted risk is below 1% (a); RG-2 represents patients whose acute MI predicted risk is between 1% and 1.5% (b); RG-3 represents patients whose acute MI predicted risk is larger than 1.5% (c). A well-calibrated estimator should have the true effect size within the 95 percent confidence interval 95 percent of times.

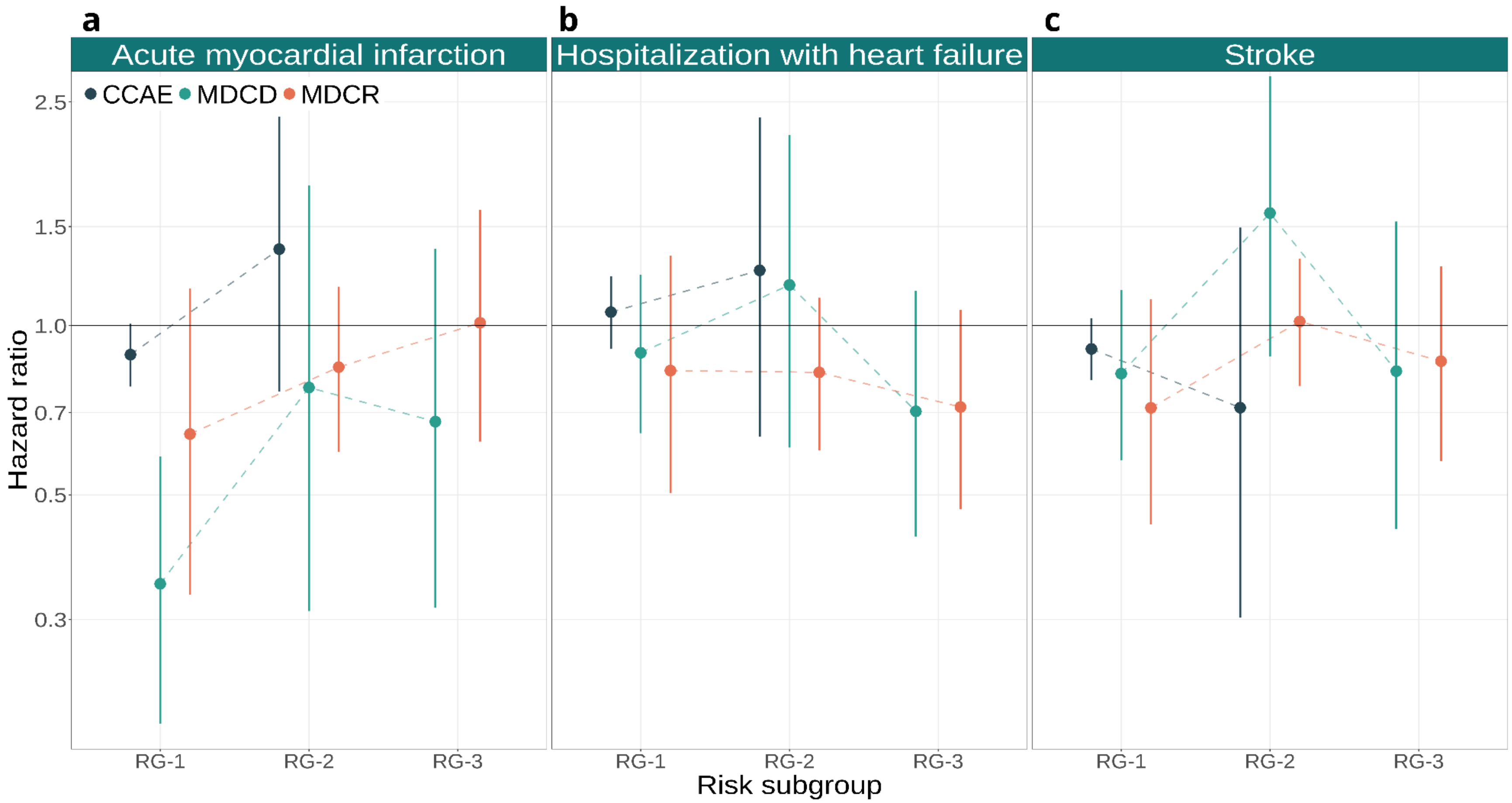

Supplementary Figure 4. Relative treatment effects for the main outcomes in patients without cardiovascular disease. Treatment effect heterogeneity in the subset of patients without cardiovascular disease for (a) acute myocardial infarction, (b) hospitalization with heart failure, and (c) stroke on the relative scale (hazard ratios) of thiazide or thiazide-like diuretics within strata of predicted acute MI risk. RG-1 represents the group of patients with acute MI risk below 1%; RG-2 represents the group of patients with acute MI risk between 1% and 1.5%; RG-3 represents the group of patients with acute MI risk larger than 1.5%. Hazard ratios estimated in CCAE, MDCD, and MDCR are represented by blue, green, and orange circles, respectively. The bars represent 95% confidence intervals. Values below 1 favor thiazide or thiazide-like diuretics, while values above 1 favor ACE inhibitors.

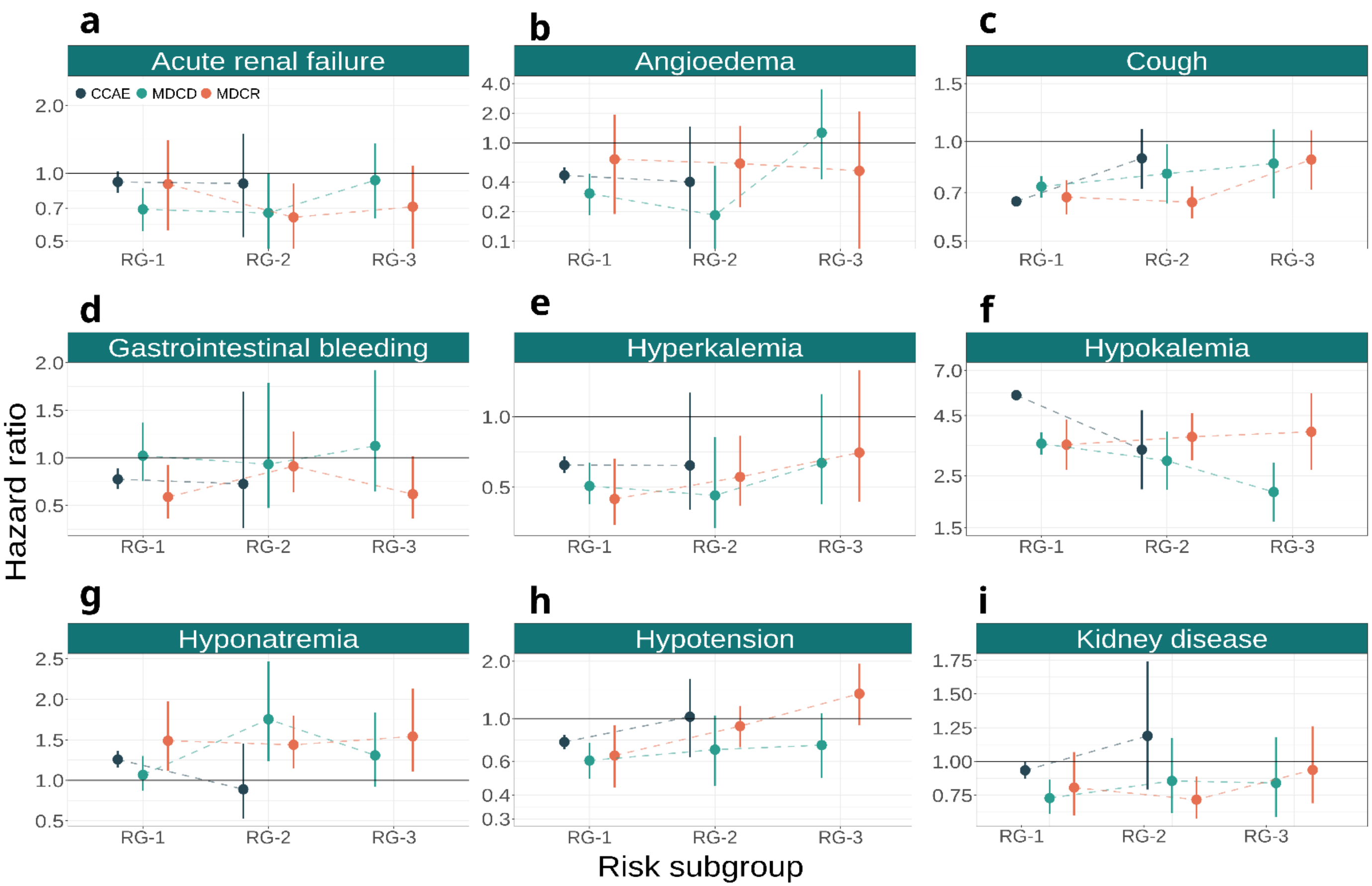

Supplementary Figure 5. Relative treatment effects for the safety outcomes in patients without cardiovascular disease. Treatment effect heterogeneity in the subset of patients without cardiovascular disease for (a) acute renal failure, (b) angioedema, (c) cough, (d) gastrointestinal bleeding, (e) hyperkalemia, (f) hypokalemia, (g) hyponatremia, (h) hypotension, and (i) kidney disease on the relative scale (hazard ratios) of thiazide or thiazide-like diuretics within strata of predicted acute MI risk. RG-1 represents the group of patients with acute MI risk below 1%; RG-2 represents the group of patients with acute MI risk between 1% and 1.5%; RG-3 represents the group of patients with acute MI risk larger than 1.5%. Hazard ratios estimated in CCAE, MDCD, and MDCR are represented by blue, green, and orange circles, respectively. The bars represent 95% confidence intervals. Values below 1 favor thiazide or thiazide-like diuretics, while values above 1 favor ACE inhibitors.

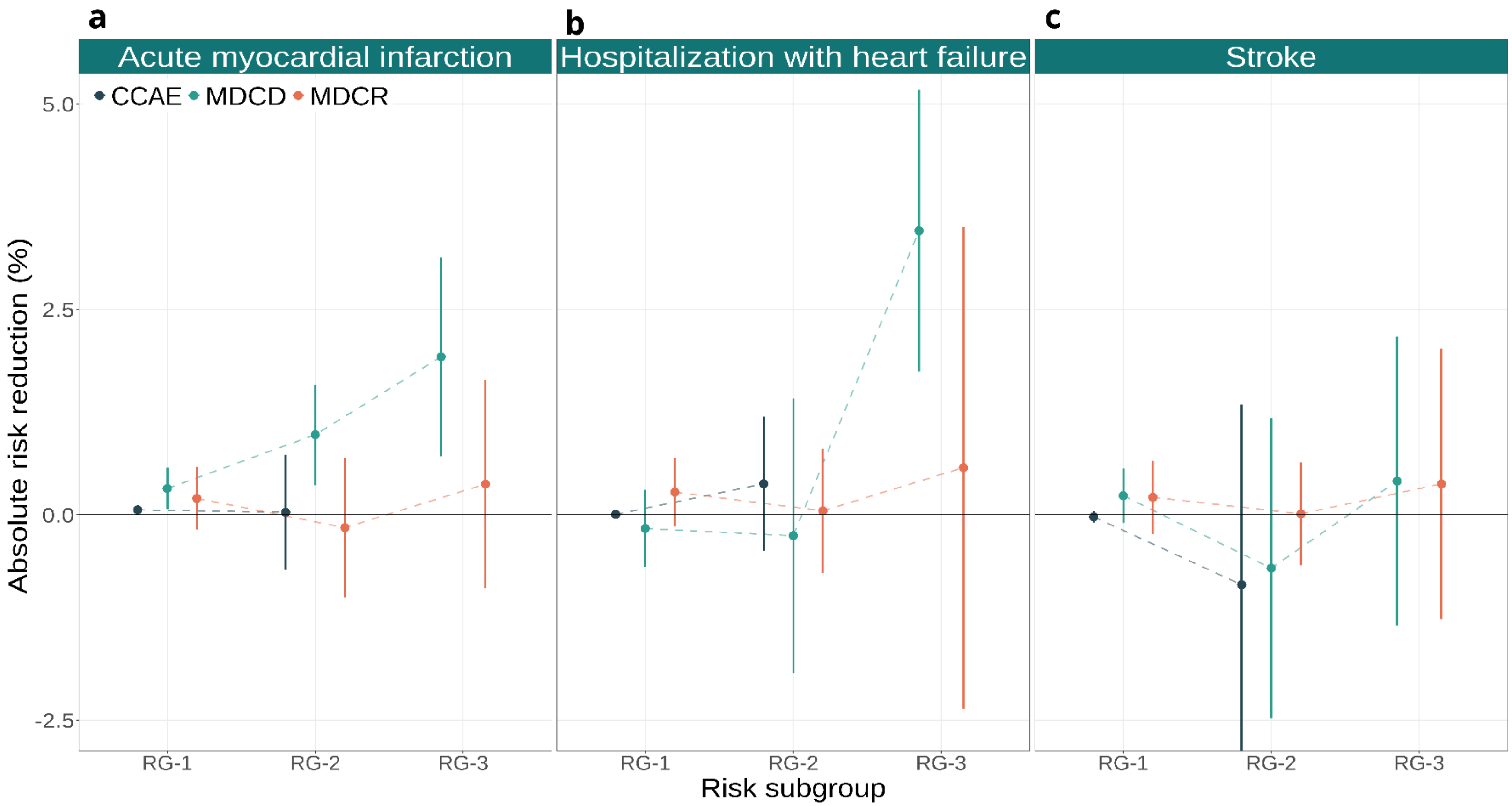

Supplementary Figure 6. Absolute treatment effects for the main outcomes in patients without cardiovascular disease. Treatment effect heterogeneity in the subset of patients without cardiovascular disease (a) acute myocardial infarction, (b) hospitalization with heart failure, and (c) stroke on the absolute scale of thiazide or thiazide-like diuretics within strata of predicted acute MI risk. RG-1 represents the group of patients with acute MI risk below 1%; RG-2 represents the group of patients with acute MI risk between 1% and 1.5%; RG-3 represents the group of patients with acute MI risk larger than 1.5%. Absolute treatment effects estimated in CCAE, MDCD, and MDCR are represented by blue, green, and orange circles, respectively. The bars represent 95% confidence intervals. Values above 0 favor thiazide or thiazide-like diuretics, while values below 0 favor ACE inhibitors.

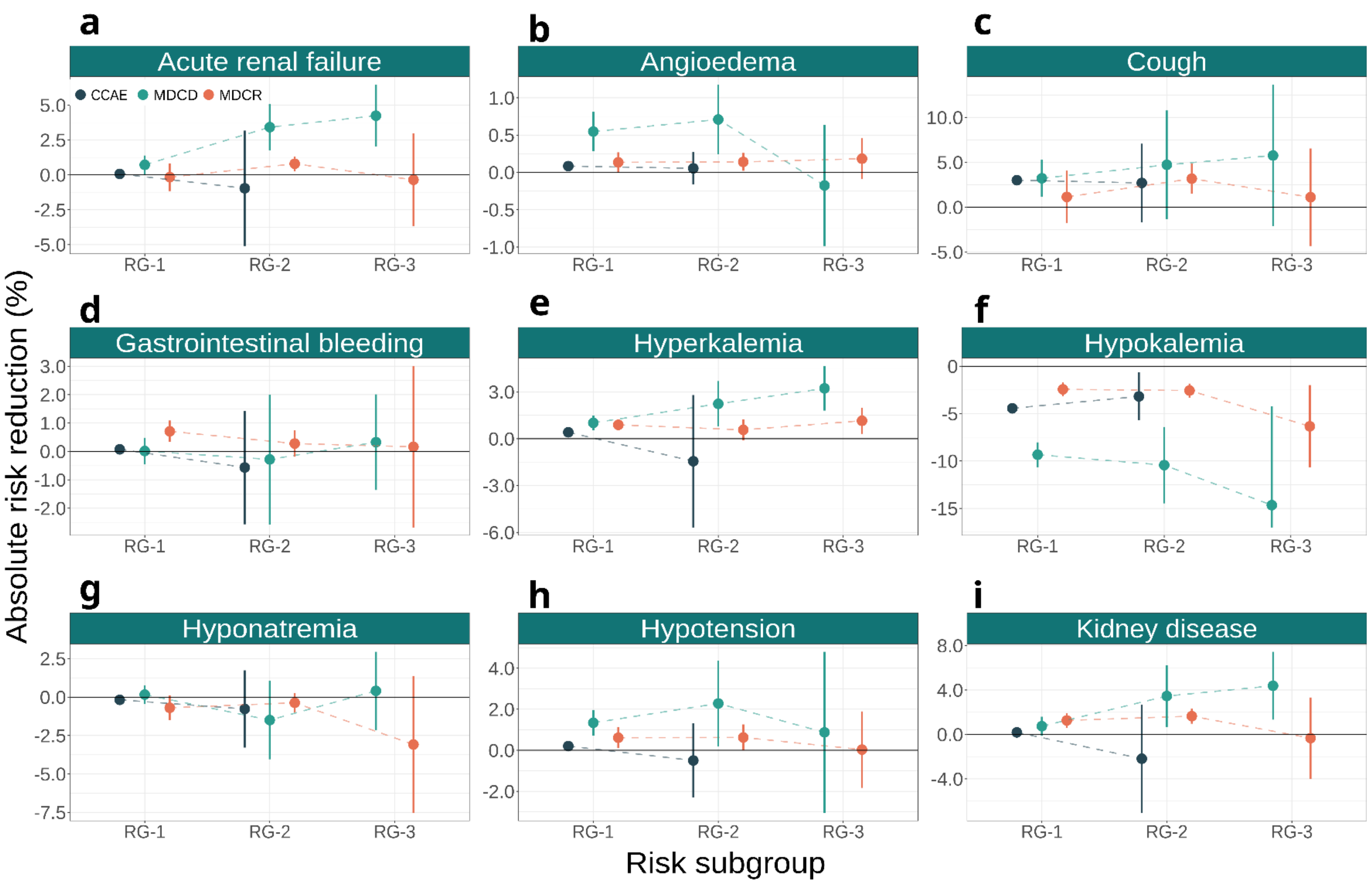

Supplementary Figure 7. Absolute treatment effects for the safety outcomes in patients without cardiovascular disease. Treatment effect heterogeneity in the subset of patients without cardiovascular disease for (a) acute renal failure, (b) angioedema, (c) cough, (d) gastrointestinal bleeding, (e) hyperkalemia, (f) hypokalemia, (g) hyponatremia, (h) hypotension, and (i) kidney disease on the absolute scale of thiazide or thiazide-like diuretics within strata of predicted acute MI risk. RG-1 represents the group of patients with acute MI risk below 1%; RG-2 represents the group of patients with acute MI risk between 1% and 1.5%; RG-3 represents the group of patients with acute MI risk larger than 1.5%. Absolute treatment effects estimated in CCAE, MDCD, and MDCR are represented by blue, green, and orange circles, respectively. The bars represent 95% confidence intervals. Values above 0 favor thiazide or thiazide-like diuretics, while values below 0 favor ACE inhibitors.

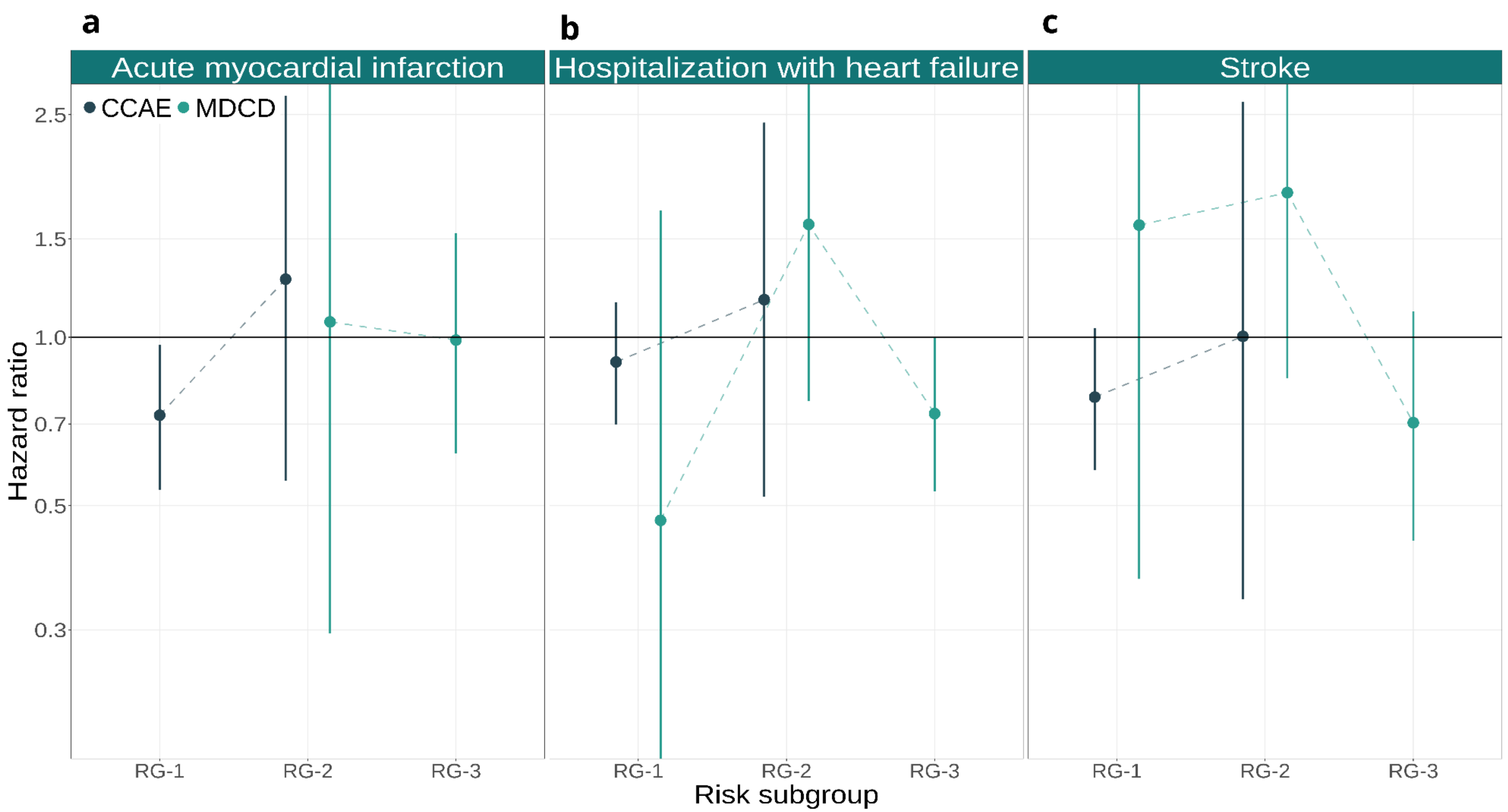

Supplementary Figure 8. Relative treatment effects for the main outcomes in patients with cardiovascular disease. Treatment effect heterogeneity in the subset of patients with cardiovascular disease for (a) acute myocardial infarction, (b) hospitalization with heart failure, and (c) stroke on the relative scale (hazard ratios) of thiazide or thiazide-like diuretics within strata of predicted acute MI risk. RG-1 represents the subgroup of patients with acute MI risk below 1%; RG-2 represents the group of patients with acute MI risk between 1% and 1.5%; RG-3 represents the group of patients with acute MI risk larger than 1.5%. Values below 1 favor thiazide or thiazide-like diuretics, while values above 1 favor ACE inhibitors. Hazard ratios estimated in CCAE and MDCD are represented by blue and green circles, respectively. The bars represent 95% confidence intervals. Results in MDCR are not presented because the majority of the patients were at risk above 1.5% for acute MI.

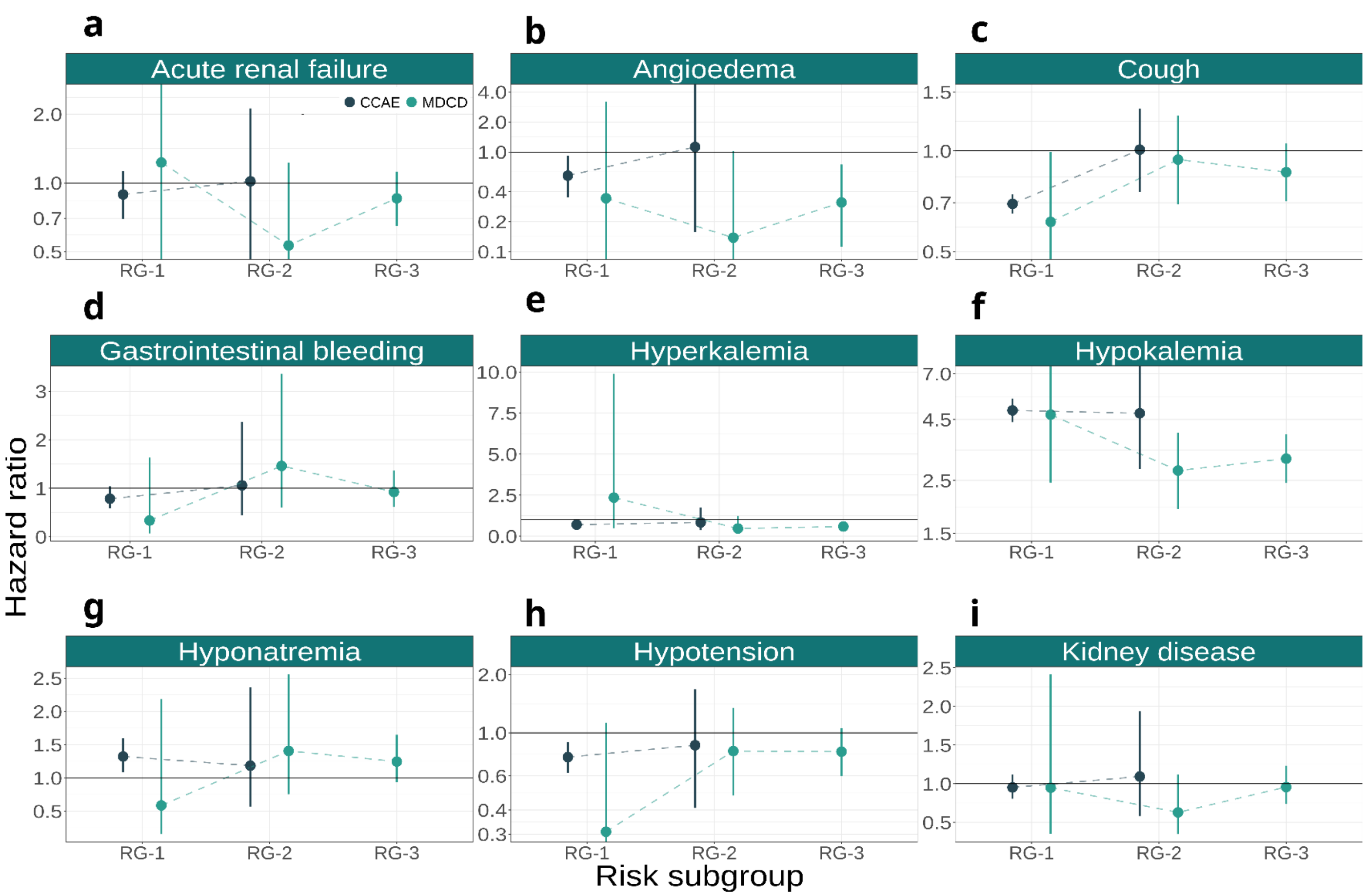

Supplementary Figure 9. Relative treatment effects for the safety outcomes in patients with cardiovascular disease. Treatment effect heterogeneity in the subset of patients with cardiovascular disease for (a) acute renal failure, (b) angioedema, (c) cough, (d) gastrointestinal bleeding, (e) hyperkalemia, (f) hypokalemia, (g) hyponatremia, (h) hypotension, and (i) kidney disease on the relative scale (hazard ratios) of thiazide or thiazide-like diuretics within strata of predicted acute MI risk. RG-1 represents the group of patients with acute MI risk below 1%; RG-2 represents the group of patients with acute MI risk between 1% and 1.5%; RG-3 represents the group of patients with acute MI risk larger than 1.5%. Hazard ratios estimated in CCAE and MDCD are represented by blue and green circles, respectively. The bars represent 95% confidence intervals. Values below 1 favor thiazide or thiazide-like diuretics, while values above 1 favor ACE inhibitors. Results in MDCR are not presented because the majority of the patients were at risk above 1.5% for acute MI.

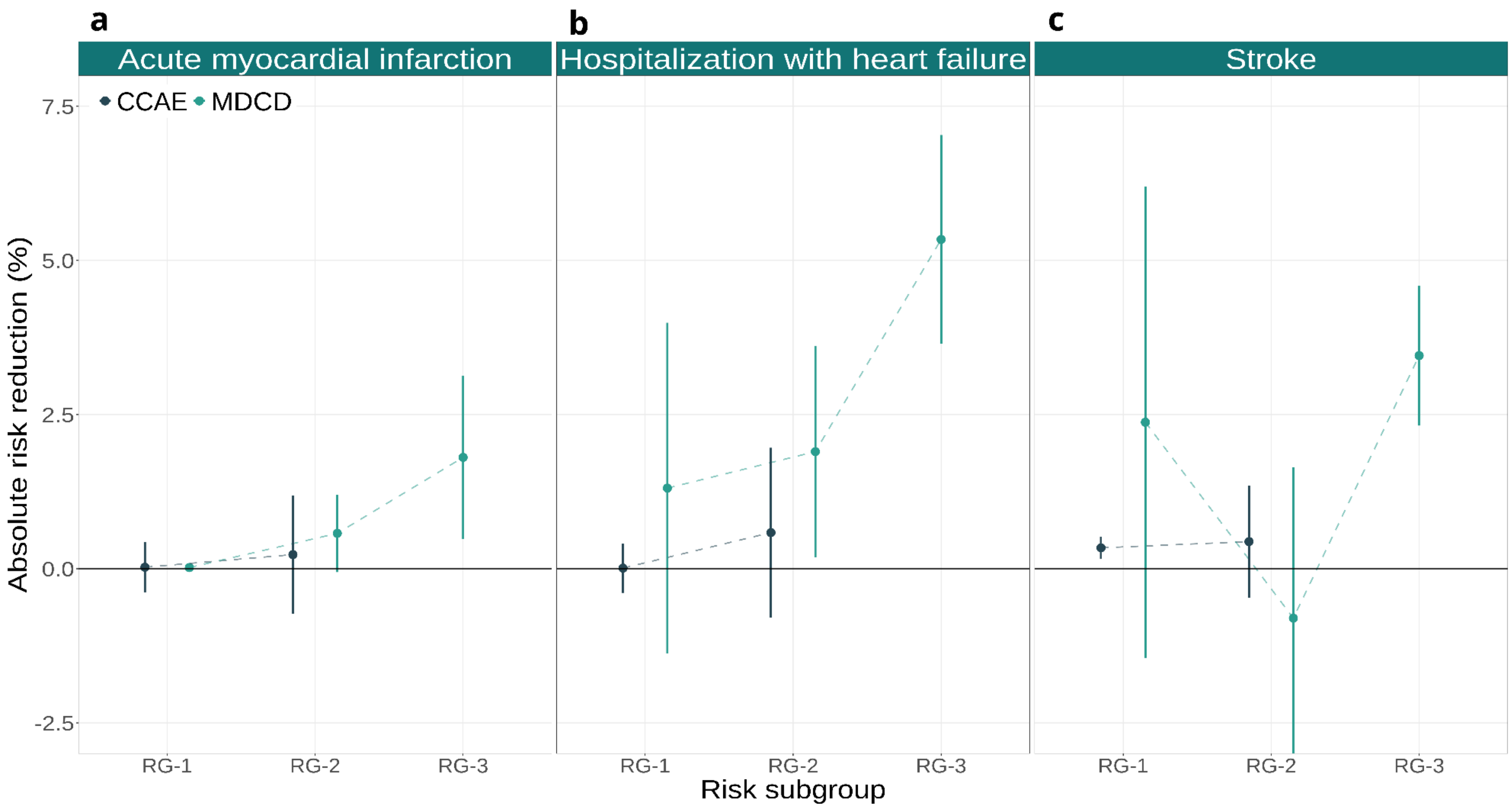

Supplementary Figure 10. Absolute treatment effects for the main outcomes in patients with cardiovascular disease. Treatment effect heterogeneity in the subset of patients with cardiovascular disease for the main outcomes of interest on the absolute scale of thiazide or thiazide-like diuretics within strata of predicted acute MI risk. RG-1 represents the group of patients with acute MI risk below 1% (a); RG-2 represents the group of patients with acute MI risk between 1% and 1.5% (b); RG-3 represents the group of patients with acute MI risk larger than 1.5% (c). Absolute treatment effects estimated in CCAE and MDCD are represented by blue and green circles, respectively. The bars represent 95% confidence intervals. Values above 0 favor thiazide or thiazide-like diuretics, while values below 0 favor ACE inhibitors. Results in MDCR are not presented because the majority of the patients were at risk above 1.5% for acute MI.

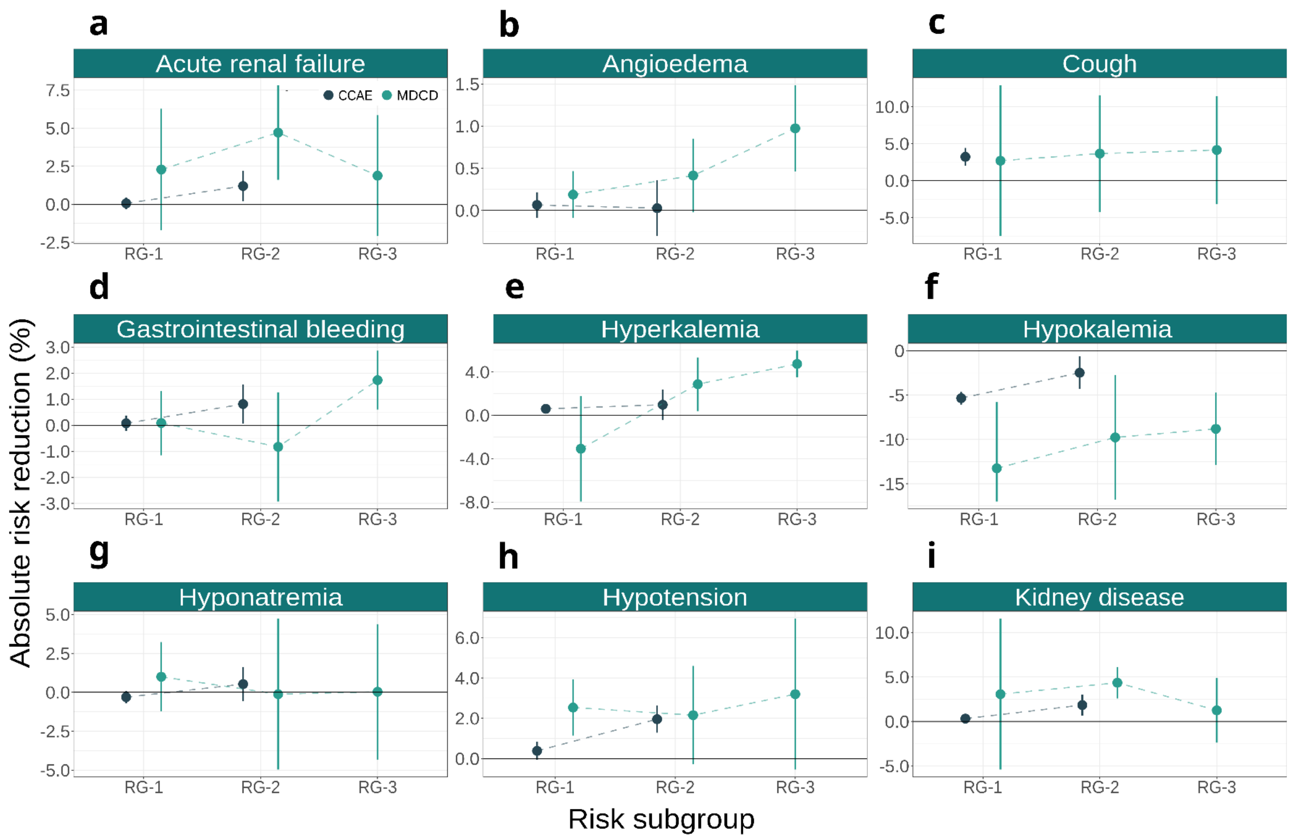

Supplementary Figure 11. Absolute treatment effects for the safety outcomes in patients with cardiovascular disease. Treatment effect heterogeneity in the subset of patients with cardiovascular disease for (a) acute renal failure, (b) angioedema, (c) cough, (d) gastrointestinal bleeding, (e) hyperkalemia, (f) hypokalemia, (g) hyponatremia, (h) hypotension, and (i) kidney disease on the absolute scale of thiazide or thiazide-like diuretics within strata of predicted acute MI risk. RG-1 represents the group of patients with acute MI risk below 1%; RG-2 represents the group of patients with acute MI risk between 1% and 1.5%; RG-3 represents the group of patients with acute MI risk larger than 1.5%. Absolute treatment effects estimated in CCAE and MDCD are represented by blue and green circles, respectively. The bars represent 95% confidence intervals. Values above 0 favor thiazide or thiazide-like diuretics, while values below 0 favor ACE inhibitors. Results in MDCR are not presented because the majority of the patients were at risk above 1.5% for acute MI.

SUPPLEMENTARY REFERENCES

1. Suchard MA, Schuemie MJ, Krumholz HM, You SC, Chen R, Pratt N, et al. Comprehensive comparative effectiveness and safety of first-line antihypertensive drug classes: a systematic, multinational, large-scale analysis. The Lancet. 2019;394:1816–26.
