# Supplementary material for: A standardized framework for risk-based assessment of treatment effect heterogeneity in observational healthcare databases
Source: NPJ Digit Med. 2023 Mar 30;6:58. doi: 10.1038/s41746-023-00794-y (PMC10060247; doi:10.1038/s41746-023-00794-y)
Supplement: Supplementary file 2 — REPORTING SUMMARY [file 41746_2023_794_MOESM2_ESM.pdf]

nature portfolio

Corresponding author(s): Alexandros Rekkas

Last updated by author(s): Mar 6, 2023

# Reporting Summary

Nature Portfolio wishes to improve the reproducibility of the work that we publish. This form provides structure for consistency and transparency in reporting. For further information on Nature Portfolio policies, see our Editorial Policies and the Editorial Policy Checklist.

## Statistics

For all statistical analyses, confirm that the following items are present in the figure legend, table legend, main text, or Methods section.

| n/a | Confirmed | |
|---|---|---|
| ☐ | ☒ | The exact sample size (*n*) for each experimental group/condition, given as a discrete number and unit of measurement |
| ☐ | ☒ | A statement on whether measurements were taken from distinct samples or whether the same sample was measured repeatedly |
| ☐ | ☒ | The statistical test(s) used AND whether they are one- or two-sided<br>*Only common tests should be described solely by name; describe more complex techniques in the Methods section.* |
| ☐ | ☒ | A description of all covariates tested |
| ☐ | ☒ | A description of any assumptions or corrections, such as tests of normality and adjustment for multiple comparisons |
| ☐ | ☒ | A full description of the statistical parameters including central tendency (e.g. means) or other basic estimates (e.g. regression coefficient) AND variation (e.g. standard deviation) or associated estimates of uncertainty (e.g. confidence intervals) |
| ☐ | ☒ | For null hypothesis testing, the test statistic (e.g. *F*, *t*, *r*) with confidence intervals, effect sizes, degrees of freedom and *P* value noted<br>*Give P values as exact values whenever suitable.* |
| ☒ | ☐ | For Bayesian analysis, information on the choice of priors and Markov chain Monte Carlo settings |
| ☒ | ☐ | For hierarchical and complex designs, identification of the appropriate level for tests and full reporting of outcomes |
| ☐ | ☒ | Estimates of effect sizes (e.g. Cohen's *d*, Pearson's *r*), indicating how they were calculated |

*Our web collection on statistics for biologists contains articles on many of the points above.*

## Software and code

Policy information about availability of computer code

| | |
|---|---|
| Data collection | Code for the extraction of the data is publicly available at https://github.com/ohdsi/Legend |
| Data analysis | Code for the execution of the study is publicly available at https://github.com/mi-erasmusmc/HteFramework. The developed R package for performing the suggested framework is publicly available at https://github.com/ohdsi/RiskStratifiedEstimation |

For manuscripts utilizing custom algorithms or software that are central to the research but not yet described in published literature, software must be made available to editors and reviewers. We strongly encourage code deposition in a community repository (e.g. GitHub). See the Nature Portfolio guidelines for submitting code & software for further information.

## Data

Policy information about availability of data

All manuscripts must include a data availability statement. This statement should provide the following information, where applicable:

- Accession codes, unique identifiers, or web links for publicly available datasets
- A description of any restrictions on data availability
- For clinical datasets or third party data, please ensure that the statement adheres to our policy

The data that support the findings of this study are available from IBM MarketScan® but restrictions apply to the availability of these data, which were used under license for the current study, and so are not publicly available. Data are however available from the authors upon reasonable request and with permission of IBM MarketScan®. Please contact Peter R. Rijnbeek with any data-related requests.

## Human research participants

Policy information about studies involving human research participants and Sex and Gender in Research.

| | |
|---|---|
| Reporting on sex and gender | This study used sex (biological attribute) captured in the IBM MarketScan® medical claims databases. Sex was included as a predictor of acute myocardial infarction risk. Effect estimates were not stratified on sex. |
| Population characteristics | Supplementary Tables 20-22. |
| Recruitment | This study has no recruitment of subjects. It is a retrospective cohort study. |
| Ethics oversight | The New England Institutional Review Board (IRB) has decided that studies conducted in the databases used here are exempt from study-specific IRB review, as these studies do not qualify as human subjects research. |

Note that full information on the approval of the study protocol must also be provided in the manuscript.

# Field-specific reporting

Please select the one below that is the best fit for your research. If you are not sure, read the appropriate sections before making your selection.

☒ Life sciences ☐ Behavioural & social sciences ☐ Ecological, evolutionary & environmental sciences

For a reference copy of the document with all sections, see nature.com/documents/nr-reporting-summary-flat.pdf

# Life sciences study design

All studies must disclose on these points even when the disclosure is negative.

| | |
|---|---|
| Sample size | Patients were extracted from three claims databases using a retrospective new-user cohort design. Cohort definitions are available in the Supplementary Material. Prediction models were developed on the 1:1 propensity score matched subset of the extracted cohorts. Effect estimates were derived on the entire extracted cohorts. |
| Data exclusions | See Supplementary Results. |
| Replication | Cohort definitions are publicly available, along with the code for data extraction. Code for the execution is publicly available. |
| Randomization | Not relevant. This is a retrospective cohort study. |
| Blinding | Not relevant. This is a retrospective cohort study. |

# Reporting for specific materials, systems and methods

We require information from authors about some types of materials, experimental systems and methods used in many studies. Here, indicate whether each material, system or method listed is relevant to your study. If you are not sure if a list item applies to your research, read the appropriate section before selecting a response.

## Materials & experimental systems

| n/a | Involved in the study | |
|---|---|---|
| ☒ | ☐ | Antibodies |
| ☒ | ☐ | Eukaryotic cell lines |
| ☒ | ☐ | Palaeontology and archaeology |
| ☒ | ☐ | Animals and other organisms |
| ☒ | ☐ | Clinical data |
| ☒ | ☐ | Dual use research of concern |

## Methods

| n/a | Involved in the study | |
|---|---|---|
| ☒ | ☐ | ChIP-seq |
| ☒ | ☐ | Flow cytometry |
| ☒ | ☐ | MRI-based neuroimaging |
